# Activation of Hippo signaling pathway mediates mitochondria dysfunction and dilated cardiomyopathy in mice

**DOI:** 10.7150/thno.62302

**Published:** 2021-08-21

**Authors:** Wei Wu, Mark Ziemann, Kevin Huynh, Gang She, Zheng-Da Pang, Yi Zhang, Thy Duong, Helen Kiriazis, Tian-Tian Pu, Ru-Yue Bai, Jing-Jing Li, Yu Zhang, Ming-Xia Chen, Junichi Sadoshima, Xiu-Ling Deng, Peter J. Meikle, Xiao-Jun Du

**Affiliations:** 1 Department of Physiology and Pathophysiology, School of Basic Medical Sciences, and Key Laboratory of Environment and Genes Related to Diseases, Ministry of Education, Xian Jiaotong University Health Science Center, Xian, China.; 2 School of Life and Environmental Sciences, Deakin University, Geelong, Victoria, Australia.; 3 Baker Heart and Diabetes Institute, Melbourne, Victoria, Australia.; 4 Center of Electron Microscopy, Xian Jiaotong University Health Science Center, Xian, China.; 5 New Jersey Medical School, Department of Cell Biology and Molecular Medicine Rutgers, New Jersey, United States of America.

**Keywords:** Hippo pathway, mitochondria, dilated cardiomyopathy, heart failure, transcriptome analysis

## Abstract

**Rationale:** Mitochondrial dysfunction facilitates heart failure development forming a therapeutic target, but the mechanism involved remains unclear. We studied whether the Hippo signaling pathway mediates mitochondrial abnormalities that results in onset of dilated cardiomyopathy (DCM).

**Methods:** Mice with DCM due to overexpression of Hippo pathway kinase Mst1 were studied. DCM phenotype was evident in adult animals but contractile dysfunction was identified as an early sign of DCM at 3 weeks postnatal. Electron microscopy, multi-omics and biochemical assays were employed.

**Results:** In 3-week and adult DCM mouse hearts, cardiomyocyte mitochondria exhibited overt structural abnormalities, smaller size and greater number. RNA sequencing revealed comprehensive suppression of nuclear-DNA (nDNA) encoded gene-sets involved in mitochondria turnover and all aspects of metabolism. Changes in cardiotranscriptome were confirmed by lower protein levels of multiple mitochondrial proteins in DCM heart of both ages. Mitochondrial DNA-encoded genes were also downregulated; due apparently to repression of nDNA-encoded transcriptional factors. Lipidomics identified remodeling in cardiolipin acyl-chains, increased acylcarnitine content but lower coenzyme Q10 level. Mitochondrial dysfunction was featured by lower ATP content and elevated levels of lactate, branched-chain amino acids and reactive oxidative species. Mechanistically, inhibitory YAP-phosphorylation was enhanced, which was associated with attenuated binding of transcription factor TEAD1. Numerous suppressed mitochondrial genes were identified as YAP-targets.

**Conclusion:** Hippo signaling activation mediates mitochondrial damage by repressing mitochondrial genes, which causally promotes the development of DCM. The Hippo pathway therefore represents a therapeutic target against mitochondrial dysfunction in cardiomyopathy.

## Introduction

Dilated cardiomyopathy (DCM) and heart failure (HF) represent major clinical and economic burdens worldwide. The central pathophysiology of DCM and HF is attenuated myocardial performance. Current consensus is that the failing myocardium suffers from energy shortage as a common mechanism in worsening of HF 1-3. This situation is aggravated by chamber dilatation, elevated wall tension and interstitial fibrosis. Cardiac ATP is mainly consumed in contraction-relaxation dynamics 1, 3. As ATP storage is extremely limited in the heart, mitochondria must operate efficiently to meet the energy demand. The failing heart exhibits mitochondrial dysfunction, featured as (i) reduction in fatty acid ß-oxidation or branched-chain amino acid (BCAA) catabolism, (ii) activation of glycolysis, (iii) excess generation of reactive oxygen species (ROS), and (iv) excess mitochondrial death signaling 1, 3. However, mitochondrial abnormalities might also be a causal factor in the pathogenesis of cardiomyopathy, such as in subjects carrying mutations of mitochondrial DNA (mtDNA)-encoded genes 4. Whereas standard drug therapy is expected to partially correct energy imbalance by lowering energy demand 1, interventions targeting mitochondrial dysfunction are currently lacking.

Studies have reported activated Hippo signaling in patients and animals with cardiomyopathies 5-9. The Hippo pathway is a highly conserved signaling cascade that controls organ size during development 10. The main signal output of the Hippo pathway is through transcriptional co-regulator yes-associated protein (YAP), which orchestrates with transcription factors like TEA-domain family member1 (TEAD1), to regulate expression of numerous target genes. The nuclear localization and transcriptional activity of YAP are suppressed following Ser^127^-phosphorylation by upstream kinases, mammalian sterile 20-like kinase1 (Mst1) and large tumor suppressor homolog (Lats) 10. Recent studies have identified a role of Hippo-YAP signaling in heart disease 6, 10-12. Whereas enhanced YAP activity facilitates post-infarct cardiac healing and regeneration 6, 12, activation of Hippo signaling by transgenic Mst1 overexpression (Mst1-TG) leads to DCM 13, 14 and interstitial fibrosis 9, 13. A similar DCM phenotype has been reported in a mouse model of cardiomyocyte-restricted YAP gene deletion (YAP-cKO) 12, 15, implying a common mechanism.

In settings of DCM and HF, it remains unclear whether mitochondrial dysfunction is the consequence of aberrant signaling mechanism(s). This possibility has been suggested by recent studies. We noticed in Mst1-TG mice, a significant downregulation of numerous nuclear DNA (nDNA)-encoded mitochondrial gene-sets 13. Using cardiomyocyte-restricted TEAD1-deletion (TEAD1-cKO) models, two independent groups have recently reported rapid onset of DCM phenotype, together with downregulation of mitochondrial genes and mitochondrial dysfunction 16-18, implying a role of TEAD1 in mitochondrial integrity and function 16-18. Being an effector in YAP/TAZ-mediated transcriptional regulation, TEAD1 also interacts with other pathways or transcriptional cofactors 19. Thus, we used the Mst1-TG model to investigate the hypothesis that activation of Hippo pathway, with YAP/TEAD1 inactivation, leads to mitochondrial dysfunction and DCM.

## Methods

### Animals

The Mst1-TG strain (*TG line 28*) of mice was generated by Yamamoto *et al* with expression of the Mst1 transgene (Stk4) driven by the cardiomyocyte-restricted α-myosin heavy chain promoter (α-MHC) 14. The DCM phenotype in the Mst1-TG model has been well documented by our previous studies 13, 14, 20. Genotyping was performed, then TG and litter mate non-TG (nTG) mice were used at young (3 to 4 weeks) and adult age (average 6 months, range from 5.5 to 7 months). The DCM phenotype was similarly present in male and female TG mice, and in the present study male nTG and TG mice were used. Animals were housed in a standard temperature-controlled environment under a 12-h light-dark cycle. All protocols and procedures used in this study were approved by local animal ethics committee and conformed to the Guide for the Care and Use of Laboratory Animals published by the National Institutes of Health.

### Echocardiography

Echocardiography was performed on mice with the Vevo2100 machine (VisualSonics, Toronto) and a MS550D transducer 13. Mice at the earliest ethically acceptable age (4 weeks) were anesthetized by isoflurane (4% for induction, 1.7% for experiment) and body temperature was monitored using a rectal temperature probe and controlled at 36-37 °C. B-mode loops of the left parasternal long-axis view of the heart were captured. Left ventricular (LV) volumes at end-diastole and end-systole were obtained, and ejection fraction (EF) was calculated. M-mode traces were obtained from short-axis 2-D view of the LV. LV dimensions at end-diastole and end-systole (LVDd, LVDs) were measured and fractional shortening (FS) was calculated [(LVDd - LVDs)/LVDd × 100%]. Thickness of anterior and posterior walls (AW, PW) of the LV was measured and LV mass was calculated as [(LVDd + AW + PW)^3^ - LVDd^3^] × 1.055. The right ventricle (RV) was visualized on the right parasternal long-axis view 13. RV dimensions at end-systole and end-diastole were measured, and fractional shortening (RVFS) was calculated. All loops and images of three cardiac cycles were analyzed in a blinded fashion and the average was used.

### Tissue collection and organ morphometry

At the end of experiments, animals were killed by anaesthetic overdose (mixture of ketamine, xylazine and atropine at 200/40/2.4 mg/kg, respectively, i.p) or under deep isoflurane anaesthesia (4%). A blood sample was collected by cardiac puncture followed by cervical dislocation. The heart was removed and the LV, RV and atria were separated, weighed and stored in -80 °C. LV tissues and plasma were used for assays. Wet weights of the kidney, liver and lungs were measured with results normalized by body weight (3-wk-old mice) or tibial length (adult mice).

### Transmission electron microscopy (EM) and quantification

Detailed EM method is provided in the supplementary material.

### Quantitative reverse transcription PCR

The detailed quantitative reverse transcription PCR (qRT-PCR) method is provided in supplementary material.

### YAP silencing by small RNA interference in H9c2 cells

The detailed method for YAP gene silencing is provided in the supplementary material.

### Immunoblotting

Protein immunoblotting was performed as previously described 9, 21. Protein was extracted from LV tissue by homogenization of tissues with lysis buffer containing phosphatase protease inhibitors. For immunoblots, protein (20-40 μg) together with molecular markers (Thermo) were separated on 4-15% mini-PROTEAN TGX-Stain free gels (BioRad, Hercules, CA, USA) and transferred onto a polyvinylidene membrane (Millipore, Billerica, MA). The membrane was blocked with 5% skim milk in Tris-buffered saline-Tween 20 and incubated overnight at 4 °C with primary antibodies against test proteins (see Table S2), followed by incubation with secondary antibody (1:8000-1:10000). Protein bands were visualized by enhanced chemiluminescence using Clarity^TM^ Western ECL Substrate (Bio-Rad), and intensity of bands was quantified with ImageJ software (version 4.5.2, Bio-Rad, Hercules, CA, USA). Normalization of test proteins was conducted using glyceraldehyde 3-phosphate dehydrogenase (GAPDH) as a housekeeping protein detected using the same membrane.

### Preparation of cytoplasmic and nuclear protein fractions and co-immunoprecipitation

Cytoplasmic and nuclear proteins were separated and extracted using commercial Nuclear and Cytoplasmic Protein Extraction Kit (Lot number P0028, Beyotime Biotechnology) according to the manufacturer's instructions. Specifically, fresh LV tissues (approximately 30 mg) were harvested, minced, washed in mixed extract solutions A and B (20:1) containing PMSF (1 mM, 4 °C). The homogenate was centrifuged at 1,500 g for 5 min (4 °C) and the supernatant was removed. The pellet was thoroughly vortexed (5 s for 3 times with intervals of 10-15 min on ice), centrifuged (15,000 g, 5 min, 4 °C) and the supernatant as cytoplasmic protein fraction was harvested. The pellet was resuspended and centrifuged (15,000 g, 5 min, 4 °C). After removal of the supernatant, the pellet was redissolved, sonicated and centrifuged (15,000 g, 10 min, 4 °C). The supernatant containing nuclear proteins was harvested.

Co-immunoprecipitation (co-IP): After adjusting the concentration of nuclear protein to 1 μg**/**μL, 40 μL as input was added with 10 μL agarose beads, incubated at 4 °C for 1 h, and centrifuged (2,500 rpm, 1 min, 4 °C). The supernatant was transferred into 2 tubes and with addition of antibody against either YAP1 or TEAD1 or with IgG. The tubes were rotated overnight at 4 °C, with the addition of 50 μl per tube protein A/G PLUS-agarose beads in the final 3 h. Samples were centrifuged (2,000 rpm, 1 min, 4 °C) and supernatant discarded. The precipitate was added with 40 μL 2× loading buffer and boiled at 95 °C for 10 min. Then immunoblotting was performed as described above. Separation of cytoplasmic and nuclear protein fractions was confirmed using GAPDH (cytoplasmic marker) and Lamin-B1 (nuclear marker).

### Determination of myocardial reactive oxygen species (ROS)

Two independent methods were applied in determination of ROS, as we previously described 21, 22. First, O^2-^ formation was estimated using OCT fresh-frozen LV sections (5 μm) incubated with dihydroethidium (DHE 5 μM, Invitrogen Co.) for 1 h at 37 °C. Images were obtained using an Olympus DP-72 digital microscopy (B&B Microscopes Ltd.) with excitation/emission at 488/610 nm. Second, cardiomyocytes were prepared by heart perfusion with enzymatic digestive solution, and pre-incubated with the chloromethyl derivative CM-H_2_DCFDA (DCF, 5 μM, ThermoFisher Scientific) for 30 min and then washed. DCF fluorescence images were obtained using a Leica TCS SP8 STED 3X confocal microscope with 40×, 1.3 NA oil immersion objective (excitation 488 nm, emission 505-530 nm) with fixed scanning parameters. An average of 160 cells of each heart were examined for intracellular distribution of DCF fluorescence.

### ATP and lactate assays

Fresh LV tissues were harvested, washed in cold PBS, frozen promptly and used on the same day for determination of content of ATP and lactate. ATP assay was performed using commercial ATP assay kits (Lot number A095-2-1, Jiancheng Bioengineering Institute, Nanjing, China) according to the manufacturer's instruction. ATP assay was based on the fluorescence signal emitted during luciferin catabolism by luciferase, with fluorescence intensity being proportional to the content of ATP. Fluorescence of standards and test samples was read using a Victor^TM^ X2 multimode plate reader (PerkinElmer) luminometer (Waltham, USA). Lactate content in heart tissues was measured by colorimetry using a commercial L-lactatic acid colorimetric assay kit (Lot number: E-BC-Ko44-M, ELAB science - BioScientific Pty. Ltd. (Sydney, Australia)). Using NAD^+^ as H^+^ receptor, LDH catalyzes the reaction of lactic acid and NAD^+^ to generate pyruvic acid and NADH, respectively. NADH was subsequently converted to NBT, which is a purple chromogenic substrate. OD values at 530 nm were measured using the Victor^TM^ X2 multimode plate reader, and the concentration of lactic acid calculated based on the standard curve. Samples were determined in duplicates and the average was reported.

### Mitochondrial lipid and amino acid profiling

The detailed method is provided in the supplementary material. In brief, LV tissues were homogenized and lipids extracted as previously described 23. As we reported previously 24, lipidomic analysis of tissue extracts or plasma samples was performed by LC ESI-MS/MS using an Agilent 1290 liquid chromatography system and an Agilent 6490 QQQ mass spectrometer. We employed source conditions identical to those previously described 24. Quantification of lipid species was determined by comparison to the relevant internal standards. In the present study that was focused on mitochondria, only 3 lipids that are largely or entirely localized in mitochondrion were analyzed, i.e. acyl-carnitine (AC), cardiolipin (CL) and ubiquinone (or coenzyme Q10, CoQ10). Lipid characterization and quantification were conducted as previously described in detail 24. Alterations in the abundance of the three lipids were expressed as relative change to that of nTG control. Myocardial content of ubiquinone in 3-wk-old mice was determined using HPLC with the SHIMADZU HPLC system (LC-2030C 3D, mobile phase: 3:7 methanol:ethanol 1 ml/min). Ubiquinone was identified by an internal standard and quantified based on a standard curve.

The extracted samples were further analyzed for specific amino acids using a separate targeted HILIC-MS/MS method. An Acquity UPLC BEH Amide 1.7 µm 2.1 × 100 mm column (Waters) was used. Chromatography was used to separate out analytes prior to mass spectrometry analysis. Results were normalized to an internal standard (L-Leucine-5,5,5-d3, Sigma Aldrich).

### Statistics

Data are presented as mean ± SEM, unless otherwise specified, with individual data points presented, whenever possible. All results were analyzed using GraphPad Prism 7 Software. Normality and equal variance were tested followed by One-way analysis of variance and unpaired Student's *t*-test for comparison between two groups. All tests were 2-sided. P < 0.05 was considered statistically significant. According to our previous studies and/or preliminary experiments on protein expression for intra‐group variation and differences between group means, we calculated the group size and found that n = 6 was sufficient to detect a difference with 95% confidence and 80% power.

### Transcriptome analysis

Transcriptome sequencing of 15-week-old nTG and TG LV tissue is described previously 13. The single-end RNA sequencing reads were obtained from NCBI SRA under accession number SRP121622. For LV tissues of 3-week-old mice, transcriptome sequencing of extracted RNA was performed by BioTree Shanghai (http://www.biotree.com.cn/). After mRNA enrichment using poly-T magnetic beads, sequencing libraries were generated using NEBNext Ultra RNA Library Prep Kit for Illumina (NEB, USA) following the manufacturer's recommendations. After quality control analysis using the Agilent Bioanalyzer 2100 system, barcoded libraries were pooled to equimolar ratios and sequenced on the NovaSeq 6000 system yielding 150 bp paired end reads.

Fastq files underwent quality trimming with Skewer 25 with a phred threshold of 10, followed by mapping to the mouse reference transcriptome with Kallisto 26. The reference transcriptome sequence was obtained from Gencode (version vM24) 27. Counts were read into R v4.0.2. Differential expression analysis was performed separately for the 3- and 15-week samples. Genes with fewer than 10 reads per sample on average were discarded. Differential analysis was performed with DESeq2 version1.28.1 28. Enrichment analysis was performed with mitch (version 1.0.6) 29 using gene sets from Reactome obtained 16th March 2020 30. Heatmaps were generated with the heatmap.2 function. Genes and gene sets with a false discovery rate (FDR) adjusted p-value <0.05 were considered statistically significant. Statistical significance of gene sets was determined by a multivariate ANOVA (MANOVA) test. FDR adjustment with the Benjamini-Hochberg method was used after DESeq2 and MANOVA tests.

## Results

### Onset of DCM following postnatal expression of the Mst1 transgene

**Figure 1:**
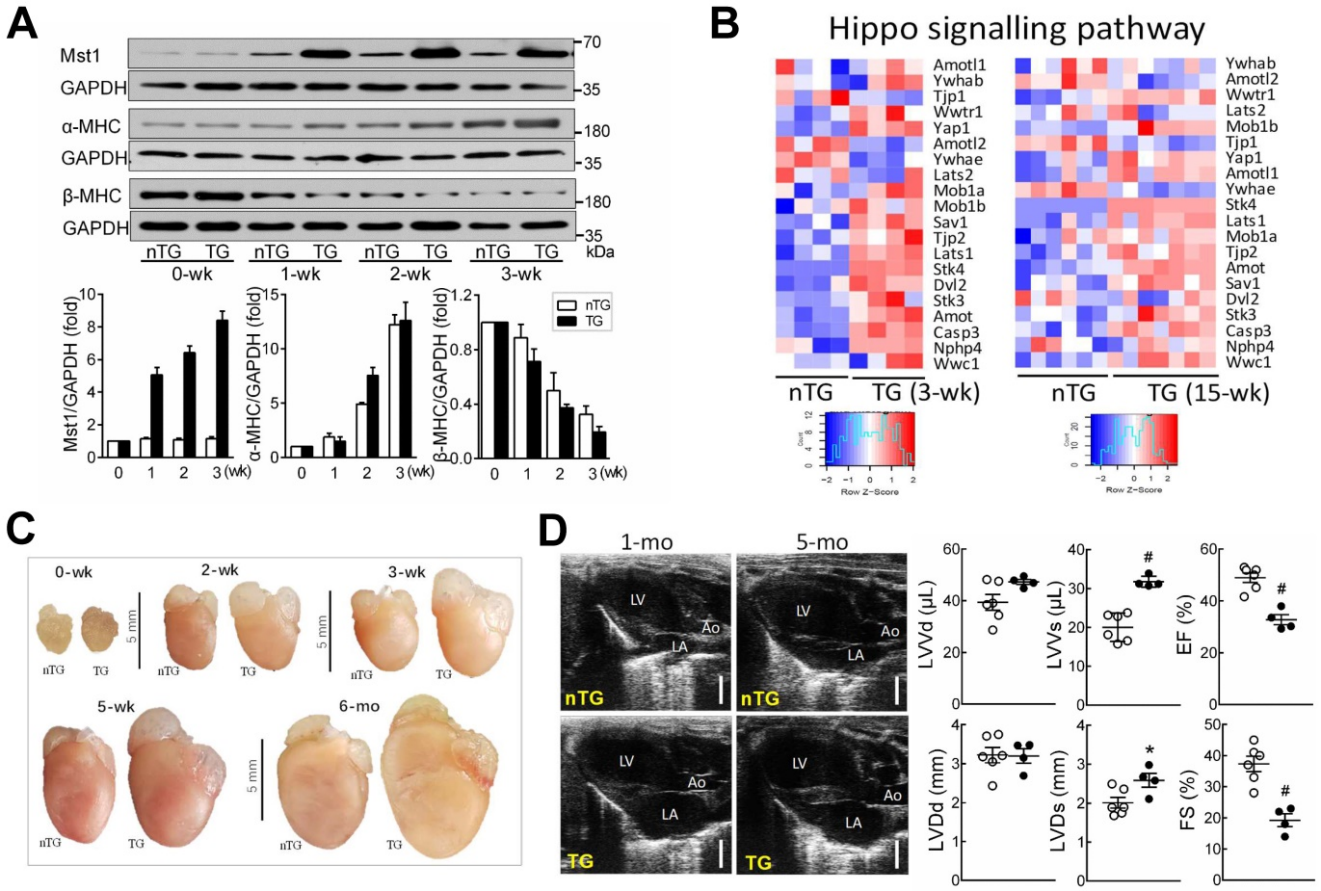
** Postnatal expression of Mst1-transgene enhanced Hippo signaling lead to early onset of DCM in Mst1-TG mice. A**, Postnatal expression of Mst1 in relation to reciprocal changes in the expression of fetal (β) and adult (α) isoforms of myosin heavy chain (MHC) by immunoblotting in LV tissues from nTG and TG mice at 0-3 weeks after birth (n = 6/group). Expression of α-MHC promoter-driven Mst1-transgene was concordant with that of α-MHC. **B**, Heatmap for expression of Hippo pathway genes by RNA sequencing in LV tissues of mice aged at 3-wk (n = 4/group) or 15-wk (n = 6-7/group). **C,** Representative images of hearts from nTG and TG mice at different ages studied. Note the enlarged size of atria and ventricles of TG mice from week-2 onwards. **D**, Representative echocardiographic end-diastolic long-axis views of the heart in nTG and TG mice aged at 1-mo and 5-mo. LA: left atrium, Ao: aorta. Bar = 2 mm. Bar graphs show B-mode loop derived LV end-diastolic or end-systolic volumes (LVVd, LVVs) and ejection fraction (EF), and M-mode traces-derived LV end-diastolic or end-systolic dimensions (LVDd, LVDs), and fractional shortening (FS) of 1-mo-old TG and nTG mice. *P < 0.05, ^#^P < 0.001 vs. nTG. Abbreviations also refer to Table S3.

The DCM phenotype in adult Mst1-TG mice has been previously described 13, 14. To test whether Hippo signaling activation could rapidly induce DCM, we determined the cardiophenotype in TG mice in relation to α-MHC promoter-driven Mst1 expression 14, 31. Mst1 protein expression was low in non-TG (nTG) hearts at 0-3 weeks, but progressively increased in TG hearts from week-1 onward (Figure 1A), in keeping with reciprocal switching from fetal (β) to adult (α) MHC isoforms. Transcriptome analysis revealed that 3-wk and adult TG hearts exhibited upregulation of Hippo pathway genes including Mst1 (Stk4), Lats1/2, scaffold proteins (Mob1, Sav1, Amot), YAP1 and transcriptional co-activator with PDZ-binding motif (TAZ, Wwtr1) (Figure 1B). Likely representing compensatory changes, downregulation occurred in Amotl2 (angiomotin-like2) and Ywhae (14-3-3), which are known to induce YAP instability and inactivation.

TG mice at 3-wks exhibited increased overall heart mass (Figure 1C) and moderate but significant increase in pulmonary or hepatic weight implying organ congestion (Figure S1). Echocardiography on 4-wk-old TG mice (relative to nTG) revealed significant reduction in LVEF or FS, and increase in LV systolic volume or dimension (Figure 1D, Table 1). There was no significant change in LV diastolic volume or dimension, nor the LV wall thickness or LV mass, implying that poor contractility of both ventricles is the primary abnormality at this early stage of DCM. In subsequent experiments, 3-wk-old and 6-mo-old mice were simultaneously studied, whenever possible, to perceive whether the observed mitochondrial abnormalities are due to activation of Hippo signaling or secondary to overt HF.

### Alterations in the cardiotranscriptome

From approximately 50,000 transcripts present in the annotation set, about 15,000 transcripts were expressed above the detection threshold (10 reads/sample). A total of 14,888 gene transcripts were identified in both age groups, and some genes were especially present in 3-wk (n = 423) or 15-wk TG hearts (n = 5,311) (Figure S2A). Relative to nTG hearts, differentially expressed genes (DEGs; false discovery rate < 0.05) accounted for 43.2% and 41.7% of genes of 3-wk and 15-wk TG hearts, respectively. Of them, equal proportions of genes were either up- or down-regulated (Figure S2B). Among DEGs in 3-wk and 15-wk TG hearts, 5,075 genes were similarly identified (Figure S2A) with 2,343 upregulated and 2,673 downregulated DEGs (Figure S2C). A filled contour plot indicated a good consistency in DEGs between both TG groups (Figure S2D) albeit 1,528 DEGs (23.1%) and 3,342 DEGs (39.7%) were only present in 3-wk or 15-wk TG groups, respectively (Figure S2A).

### Diverse changes in mitochondrial/metabolic and fibrotic genes

**Figure 2:**
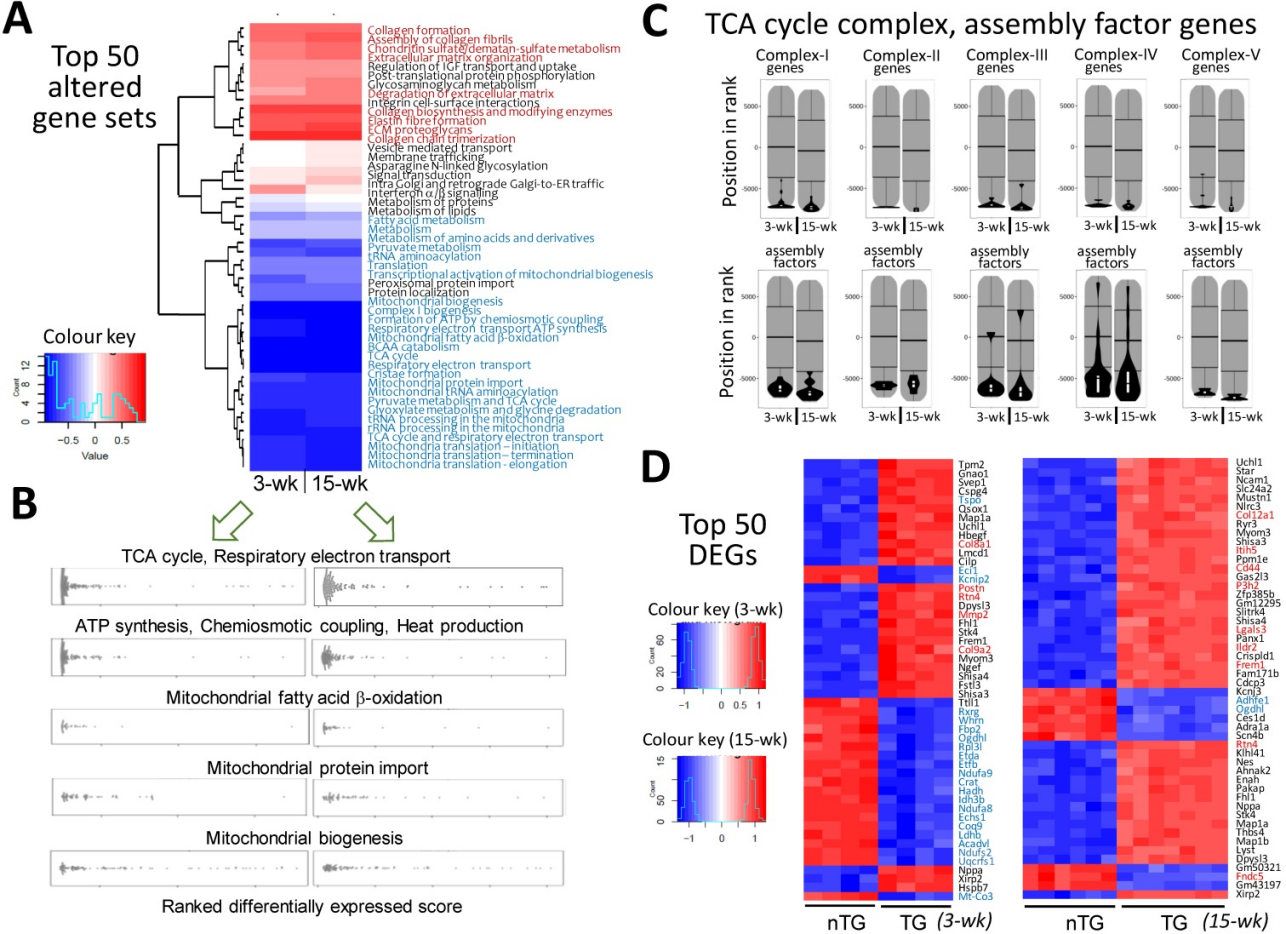
** Alterations in transcriptome and selected mitochondrial pathways by RNA-sequencing in Mst1-TG hearts.** RNA-seq data were collected from LV tissues of mice aged at 3-wk (n = 4/genotype) or 15-wk (nTG n = 6, TG n = 7). **A**, Heatmap for top-50 dysregulated gene sets by P-values in TG relative to nTG group. Names of gene-sets in red color are fibrogenesis gene sets and blue color denotes mitochondria-related gene sets. **B**, Beeswarm plots of individual gene sets related to mitochondrial metabolism or biogenesis. Note the similar changes in transcriptome in TG hearts at both ages studied. **C**, Violin-plots of genes of mitochondrial tricarboxylic acid (TCA) cycle complexes and assembly factors of both age groups. **D**, the top-50 dysregulated genes (DEG), identified by smallest P-values, relative to respective nTG groups. Red or blue color indicates genes related to either fibrosis or mitochondrion.

Pathway enrichment analysis detected approximately 1,300 gene-sets, and 18~20% of them were differentially expressed in TG hearts. More gene-sets were downregulated than upregulated (128 vs. 98 for 3-wk TG; 199 vs. 61 for 15-wk TG). As summarized in Figure 2A, the top-50 altered gene sets in TG hearts, by smallest P-values, consisted of upregulated gene-sets for fibrogenesis, but numerous suppressed mitochondrial gene-sets, including tricarboxylic acid (TCA) cycle, electron transport, biogenesis, super-complex formation/assembly, cofactor biosynthesis, fatty acid ß-oxidation, BCAA catabolism, and protein importing complex (Figure 2A-B; Figure S3, Figure S4, Figure S5). These changes were highly consistent between 3-wk and 15-wk TG groups. Notably, genes encoding TCA-cycle complexes and complex assembly co-factors were profoundly downregulated in TG hearts (Figure 2C). Of the top-50 DEGs in 3-wk TG hearts, almost all downregulated genes (20/21) were mitochondria-related (Figure 2D). Upregulation of fibrotic gene-sets was also evident in TG hearts of both age groups (Figure 2A, Figure S6) and several fibrotic genes appeared in top upregulated DEGs (Figure 2D), including galectin-3, CTGF and reticulon-4 (Rtn4) that are also YAP-target genes (Figure S6A) 13, 14. However, interstitial fibrosis by histology was only observed in adult TG hearts (Figure S6B). By Reactome enrichment analysis, expression of other gene sets was comparable between nTG and TG groups of both ages (Figure S7).

### Ultrastructural abnormalities of mitochondria in TG hearts

**Figure 3:**
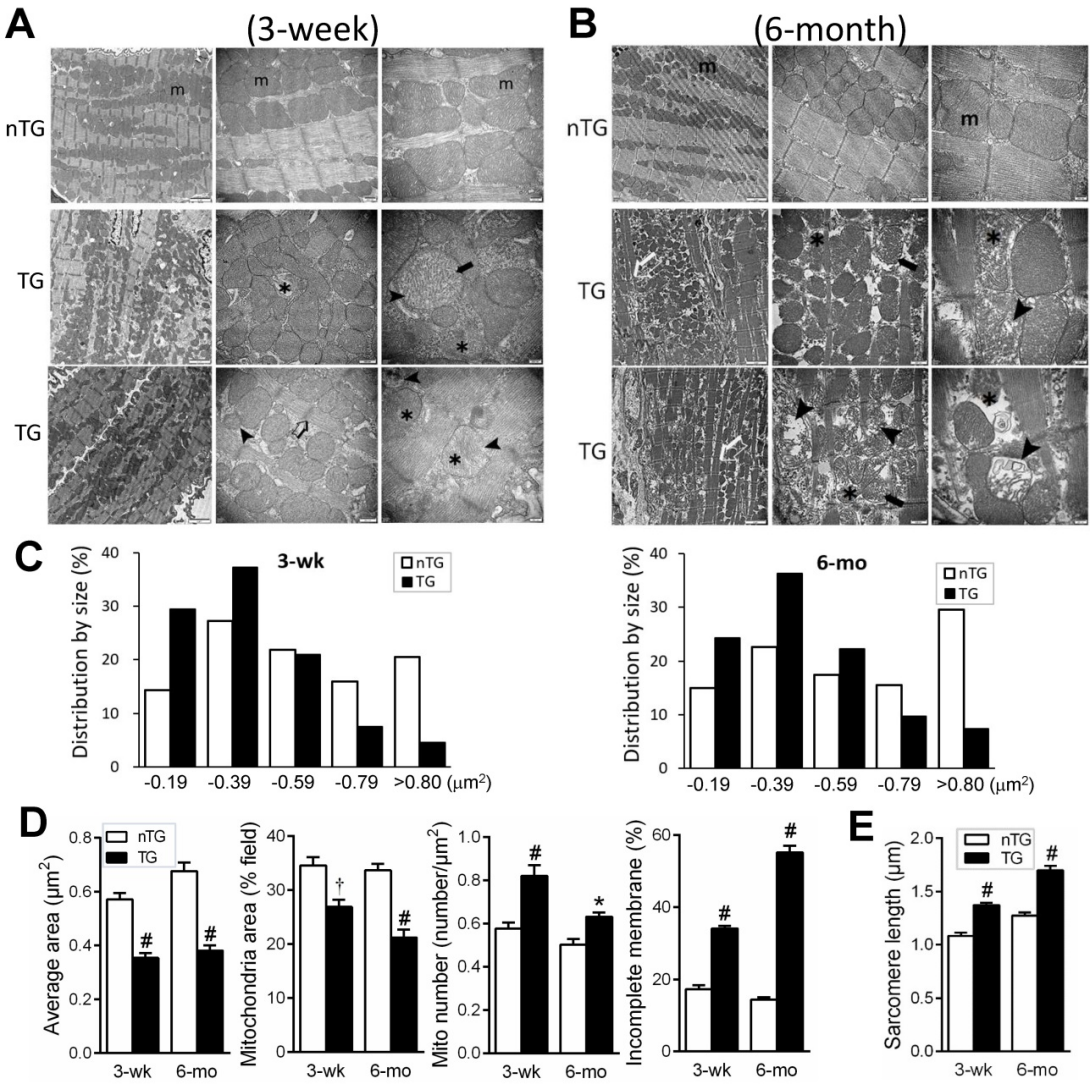
** Electron microscopic (EM) images of mouse myocardium and quantitative measures of mitochondria and sarcomeres.** Representative EM images of the LV myocardium of 3-wk (**A**) or 6-mo (**B**) nTG and TG mice (n = 3 hearts per genotype per age). From left to right: magnification at 4,000, 10,000 and 30,000, respectively. **m**: mitochondria; ➤: disruption and dissolution of cristae; ➨: mitochondrial swelling; ✱: damaged mitochondria with incomplete outer-membrane. Note the thinning and elongation of sarcomeres of TG myocardium (➯). **C**, Quantitative measures of the size and density of mitochondrion (results from 1000 to 3000 measures per heart sample). **D**, Distribution of mitochondrial size of nTG and TG mouse hearts, and the ratio of mitochondria with incomplete outer-membrane (results from 210 to 590 individual mitochondria per heart sample). **E**, Sarcomere length of nTG and TG mice. Data were from 8-10 images per heart sample with 30-40 measures per image. *P < 0.05 and ^#^P < 0.001 vs. age-matched nTG.

EM images were acquired from the LV of nTG and TG mice at 3 weeks and 6-mo of age, respectively (Figure 3). In 3-wk TG hearts, alterations in the shape and size of mitochondria were evident. Quantitative analysis showed increased mitochondrial density by 25%, particularly the fraction of small mitochondria (Figure 3C), whilst the average size was reduced by 40%. Other abnormalities included disordered alignment, swelling, partial cristae dissolution, and incomplete outer-membrane (Figure 3A, D). In 6-mo TG hearts, mitochondrial pathology was more severe (Figure 3B), exhibiting aggregation, loss of larger-sized mitochondria (Figure 3C), reduced average size (Figure 3D), and increased mitochondria with disrupted outer-membrane (Figure 3D). TG hearts exhibited thinning of myofibrils and elongated sarcomere length, indicative of DCM, which was more severe in 6-mo than 3-wk TG hearts (Figure 3E).

### Aberrant expression of mitochondrial marker proteins

**Figure 4:**
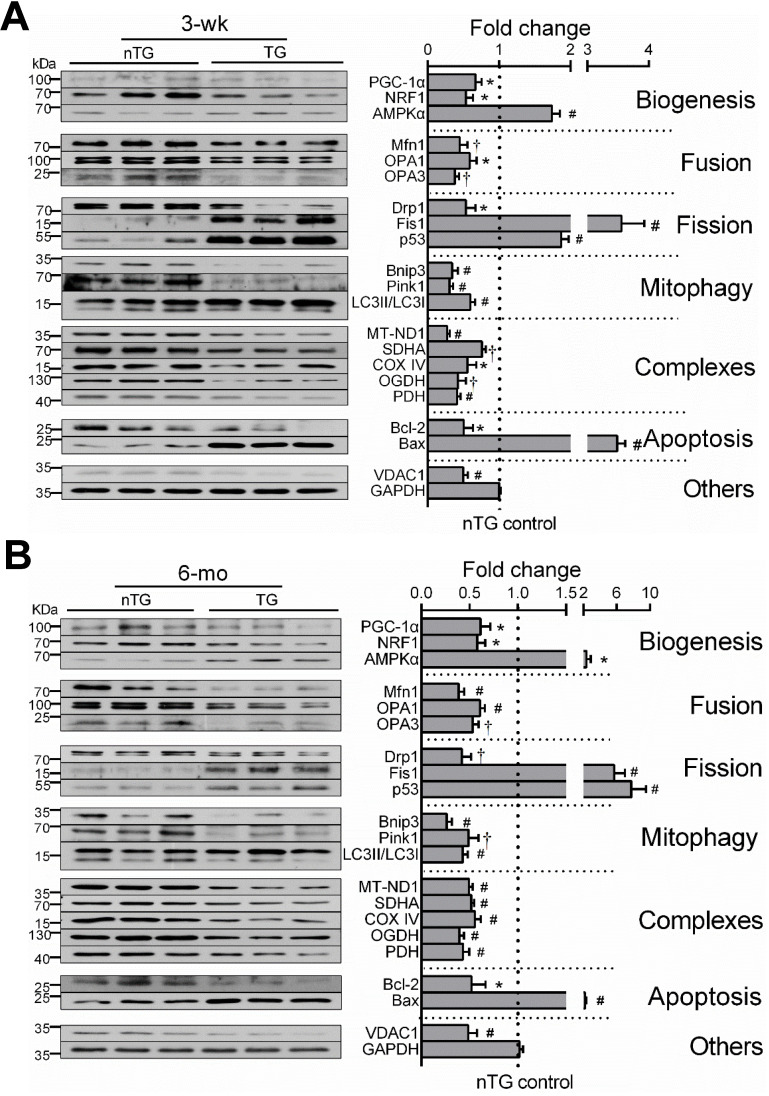
** Expression of mitochondrial marker proteins in nTG and TG hearts.** Immunoblotting was performed on protein prepared from LV tissues of 3-wk (**A**) and 6-mo (**B**) mice. Proteins tested were representative markers for mitochondrial biogenesis, fusion, fission, mitophagy, respiratory complexes I-III or TCA enzymes, apoptosis and transporter VDAC1. Protein expression was normalized by house-keeping protein GAPDH, of which expression level was similar among groups and was exposed together with marker proteins on the same membrane. For each data set, n = 6/group, *P < 0.05, †P < 0.01, and ^#^P < 0.001 vs. nTG. Abbreviations also refer to Table S3.

By immunoblotting of LV extracts, we determined protein expression of selected mitochondrial markers for complexes, turnover, mitophagy, apoptosis or VDAC1 as a pivotal transporter, and GAPDH as a housekeeping protein (Figure 4). Based on class-changes in protein markers, in 3-wk and 6-mo TG hearts, mitochondrial biogenesis signaling via PGC-1α and NRF1 was suppressed whilst another biosynthesis activator AMPKα was upregulated. Proteins involved in mitochondrial fusion or mitophagy were significantly reduced whilst fission proteins increased (Figure 4). VDAC1 abundance was 50% lower. All these changes were comparable between TG groups of both ages. Collectively, changes in protein markers not only validated our observations by RNA-seq, but also add to ultrastructural and transcriptome datasets, suggesting mitochondrial damage and dysfunction.

### Perturbed mitochondria-signature lipids

**Figure 5:**
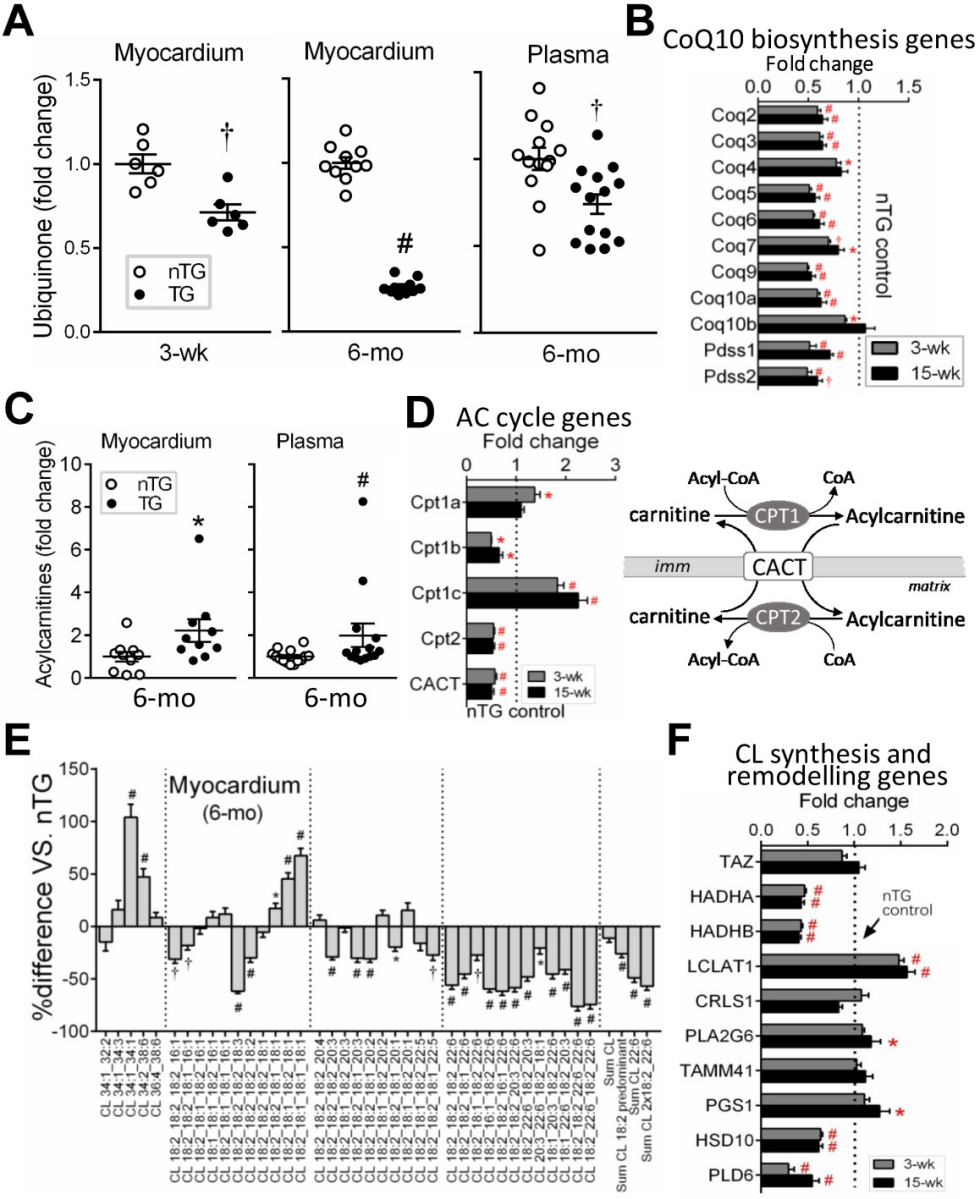
** Changes in mitochondria-specific lipids by lipidomics in TG hearts.** Lipid analysis by lipidomics was conducted in extracts of LV tissues (nTG n = 10; TG n = 11) or plasma (both n = 14) from 6-mo-old mice. **A**, Ubiquinone (CoQ10) levels in the LV myocardium and plasma of 6-mo-old mice by lipidomics. Ubiquinone was also determined in 3-wk-old mice (n = 6 each) by HPLC. **B**, Suppressed CoQ10 biosynthesis pathway genes by RNA-seq in TG hearts (n = 4/group for 3-wk and n = 6-7/group for 15-wk). **C**, Content of acylcarnitine (AC) in LV myocardium or plasma of 6-mo-old nTG and TG mice with TG results expressed as fold-changes of nTG values. **D**, Altered expression of genes encoding enzymes for acylcarnitine turnover (carnitine palmitoyltransferases, CPT) and transport (carnitine-acylcarnitine translocase, CACT). Data were from RNA-seq. **E**, Relative changes in cardiolipin (CL) species with different acyl-chains. Results presented as changes in TG relative to nTG groups. **F**, Expression of individual genes involved in CL biosynthesis or acyl-chain modifications. Data were from RNA-seq of 3-wk or 15-wk mice. For all panels, *P < 0.05, †P < 0.01, #P < 0.001 vs. nTG. Abbreviations also refer to Online Table S3.

Lipids constitute 40% of mitochondria by weight 32. By lipidomics of LV lipid extracts of 6-mo-old mice, changes in mitochondrial phospholipids, ubiquinone (i.e. CoQ10), acylcarnitine and cardiolipin (CL), were analyzed. CoQ10 is a pivotal cofactor in the electron transport chain 33, 34. Relative to respective nTG values, CoQ10 content was lower by 25% in 3-wk and by 75% in 6-mo TG hearts, together with a lower plasma CoQ10 level in 6-mo TG mice (Figure 5A). RNA-seq in both TG groups showed downregulation of CoQ10 biosynthesis pathway genes like Coq2~9 and Pdss1/2 (Figure 5B).

Cardiac and plasma levels of total acylcarnitine were higher in 6-mo TG mice compared to nTG controls (Figure 5C). Mitochondrial entry of fatty acids is mediated through transmembrane carnitine-acylcarnitine cycle driven by CPT1/2 (carnitine palmitoyltransferase) and CACT (carnitine-acylcarnitine translocase) (Figure 5D). While CPT1c expression showed a 2-fold increase, expression of CPT1b, CPT2 and CACT were suppressed in both TG groups as determined by RNA-seq (Figure 5D).

In 6-mo TG hearts, total CL content was marginally lower (P = 0.051), but CL fatty-acyl-chain species differed significantly versus nTG: CL species rich in linoleic acids (18:2) or docosahexaenoic acid (22:6) were reduced by 26% and 49%, respectively (Figure 5E), while CL species rich in saturated or monosaturated fatty-acyl-chains were higher. Typically having 4 fatty-acyl-chains, CL with 2 fatty-acyl-chains (i.e monolysocardiolipin, MLCL) undergoes fatty-acyl-chain modifications that involve enzymes like MLCL transacylase (Tafazzin, TAZ), acyl-CoA:Lysocardiolipin acyltransferase-1 (ALCAT1) and MLCL acyltransferase-1 (MLCL AT-1). RNA-seq revealed unchanged expression of CL synthesis genes (TAZ, CRLS), but diverse changes in expression of genes for fatty-acyl-chain modifications in TG hearts (Figure 5F).

### Dysfunction in mitochondrial metabolism

**Figure 6:**
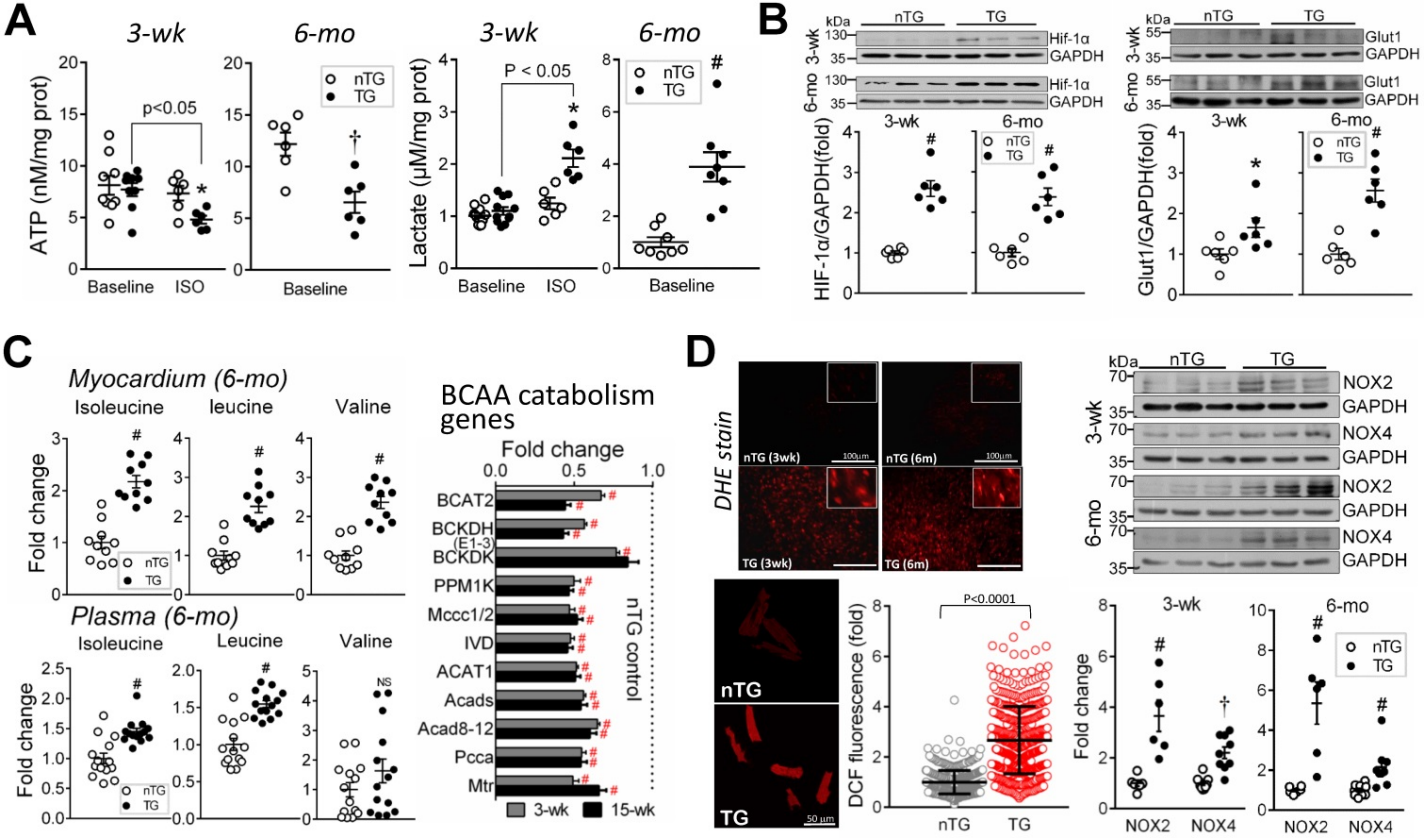
** Mitochondrial dysfunction in TG hearts. A**, Myocardial content of ATP (by luciferase chemiluminescence assay) and lactate (by commercial assay kit) in freshly prepared LV tissues from 3-wk and 6-mo mice (n = 6-9/group). Tissues were also collected from 3-wk nTG and TG mice 3-min post-injection with the β-adrenergic agonist isoproterenol (ISO, 2 mg/kg, i.p.) to enhance cardiac energy expenditure. **B**, Protein expression of the metabolic regulator hypoxia-induced factor-1α (HIF-1α) and glucose transporter protein type-1 (Glut1) of nTG and TG mice at 3-wk and 6-mo (n = 6/group). **C**, Changes in concentrations of branched-chain amino acids (BCAA, by metabolomics) in LV tissues (n = 10/group) or plasma (n = 14/group) from 6-mo-old nTG and TG mice. Shown also are RNA-seq derived BCAA catabolism genes in 3-wk and 15-wk TG relative to nTG hearts. **D**, Measurement of oxidative stress by DHE histofluorescent staining (red color, inserts showing details) of LV myocardium or by DCF staining (red color) of isolated cardiomyocytes of 6-mo-old nTG and TG mouse hearts (3 hearts per genotype with an average of 160 cells analyzed per heart; results are mean ± SD). Protein expression of NADPH oxidases, NOX2 and NOX4 (n = 6/group) in 3-wk and 6-mo nTG and TG hearts. For all data sets, *P < 0.05, †P < 0.01 and ^#^P < 0.001 vs. respective nTG. Abbreviations also refer to Table S3.

Mitochondrial metabolism was assessed by determining ATP (by luciferase chemiluminescence assay of freshly harvested LV tissues), lactate (by colorimetry), BCAA (by metabolomics), metabolic regulator HIF-α, and ROS generation. A reduction in ATP content at baseline, typically seen in advanced failing heart 1, 3, was evident in 6-mo-old (-50%, P < 0.01), but not in 3-wk-old TG mouse hearts (Figure 6A). To augment energy demand, we then treated 3-wk-old mice with β-adrenergic agonist isoproterenol (ISO, 2 mg/kg, i.p.), which promptly increased heart rate (456 ± 12 vs. 383 ± 17 beats/min, P < 0.01, n = 5). ISO-challenge showed no effect in nTG hearts, but reduced ATP content in TG hearts by about 30% (Figure 6A). Lactate content was unchanged in 3-wk TG at baseline, relative to respective nTG, but higher in 6-mo TG at baseline or in ISO-challenged 3-wk TG mice (Figure 6A). HIF-1α is a key metabolic regulator that inhibits aerobic metabolism and promotes glycolysis 35. In 3-wk and 6-mo TG hearts, HIF-1α protein level and its target glucose uptake1 (Glut1) were increased versus nTG (Figure 6B).

BCAA (isoleucine, leucine, valine) are almost entirely catabolized in mitochondria, and tissue BCAA accumulation implies mitochondrial dysfunction 36. By amino acid profiling of LV myocardium and plasma in 6-mo-old mice, tissue contents of all BCAAs were 2-fold higher when expressed in absolute, fold-change (Figure 6C), or percentage of total amino acids (all P < 0.00001). Plasma levels of isoleucine and leucine were 50% higher in TG versus nTG (P < 0.001). RNA-seq revealed pronounced downregulation of BCAA-catabolism genes in TG hearts of both age groups (Figure 6C).

Enhanced ROS generation was indicated by DHE fluorescent staining in the LV of 3-wk and 6-mo TG mice and by quantitative intracellular DCF fluorescence intensity of 6-mo-old TG mouse hearts, along with increased protein levels of NADPH oxidases, NOX2 and NOX4 (Figure 6D).

### Alterations in YAP nuclear localization, YAP/TAZ-TEAD1 interaction, and expression of YAP-target genes

**Figure 7:**
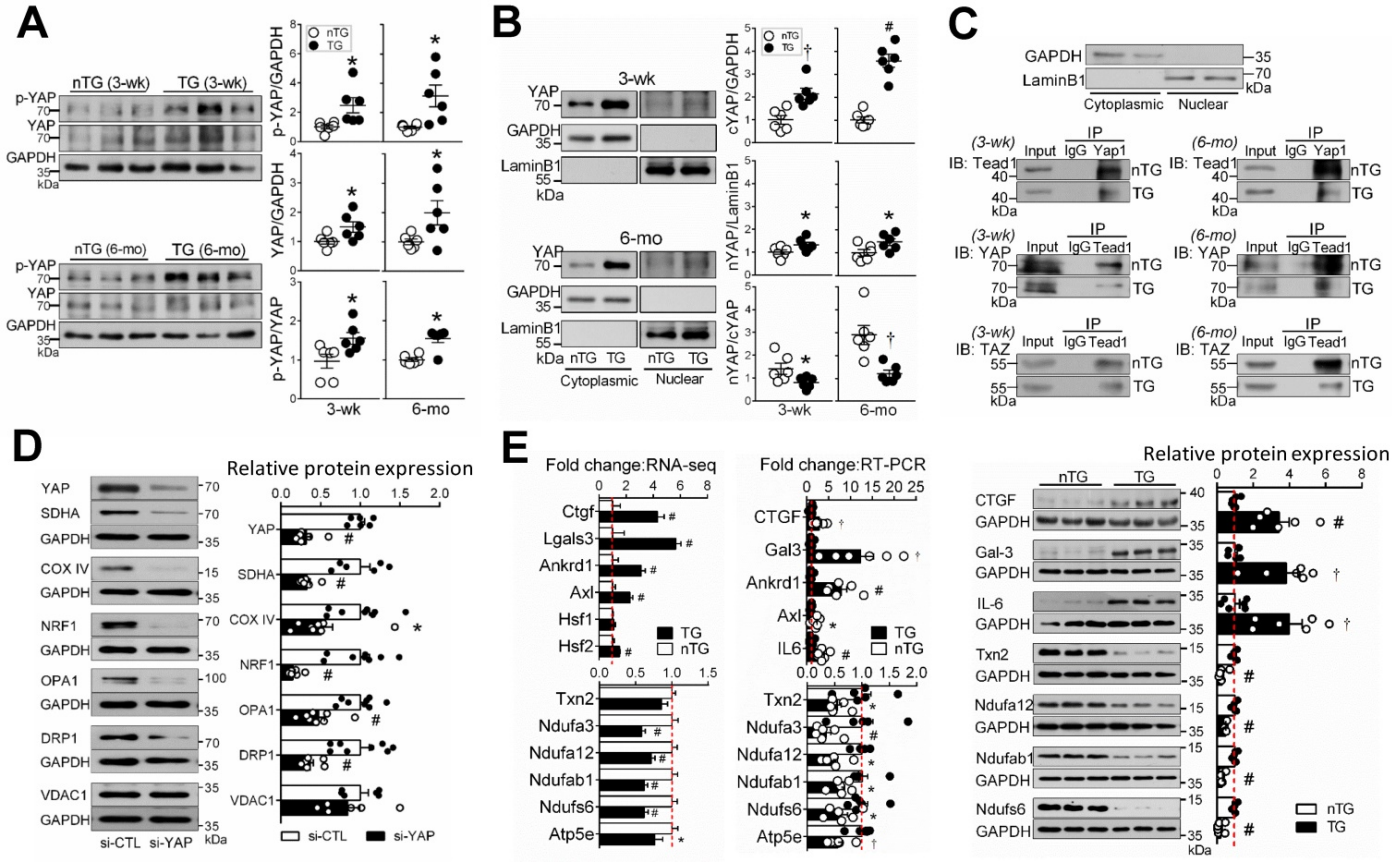
** Hippo signaling activation inhibits YAP transcriptional activity and expression of target genes. A,** Immunoblotting of YAP and Ser^127^-phospho YAP (p-YAP) in LV tissues of 3-wk and 6-mo nTG and TG mice (n = 6/group). **B,** Immunoblotting of YAP in cytoplasmic (cYAP) and nuclear (nYAP) fractions of LV tissues of 3-wk and 6-mo TG mice (n = 6/group). GAPDH and LaminB were used as cytoplasmic and nuclear markers, respectively. **C,** Representative immunoblotting (IB) images of markers for cytoplasmic (GAPDH) or nuclear proteins (Lamin-B), and co-immunoprecipitation of YAP1 or TEAD1 of nuclear proteins to determine molecular interactions. The experiments were repeated 3 times. **D**, Protein expression levels of YAP, mitochondrial marker proteins and GAPDH in H9c2 cells transfected with YAP siRNA (si-YAP) or control siRNA (si-CTL) for 48 h. 6-8 assays per group. **E**, Validation of selected YAP-target genes by qRT-PCR and immunoblotting in LV tissues of 6-mo nTG and TG mice (both n = 6/group). For all panels, *P < 0.05, †P < 0.01, ^#^P < 0.001 vs. respective nTG group. Abbreviations also refer to Table S3.

YAP protein abundance was increased in 3-wk and 6-mo TG hearts (Figure 7A), in keeping with change in gene expression (Figure 1A), likely representing a compensation to the loss of its activity when Mst1 is overexpressed. In TG hearts, there was even greater increase in Ser^127^-phospho-YAP (pYAP) yielding a higher pYAP/YAP ratio, suggestive of YAP inactivation. Relative to nTG values, increase of cytoplasmic-YAP (cYAP, by 2~4.5-fold) was much greater than that of nuclear-YAP (nYAP, by 30-40%), yielding a lower nYAP/cYAP ratio (Figure 7B). Furthermore, co-immunoprecipitation of nuclear protein showed attenuated physical interaction of nuclear YAP/TAZ and TEAD1 (Figure 7C). To further confirm the role of YAP inactivation in the loss of mitochondrial protein abundance, H9c2 cells were transfected with YAP-siRNA, which lowered YAP protein abundance by about 70% at 48 h (Figure 7D). This was associated with reduced expression of selected mitochondrial marker proteins, except VDAC1 (Figure 7D), similar to that seen in Mst1-TG hearts.

Enrichment analysis was performed on YAP-target genes according to publicly available ChIP‐seq data. Of YAP-targets, 37% (3-wk) and 39% (15-wk) genes were downregulated, and mitochondrial genes accounted for a substantial fraction, with percentages higher in 3-wk than 15-wk TG groups (Figure S8). These genes included enzymes for the biosynthesis of CoQ10 (Pdss2), Enoyl-CoA (Echdc2/3) or Acyl-CoA (Acta2), mitochondrial protein transporters (Figure S5) or mitoribosome proteins. To validate RNA-seq findings, we determined expression of YAP target genes by qRT-PCR (11 selected genes) and by immunoblotting (7 selected proteins). Changes in expression levels of these targets were consistent with RNA-seq data (Figure 7E).

### Downregulation of mitochondrial DNA (mtDNA)-encoded genes

**Figure 8:**
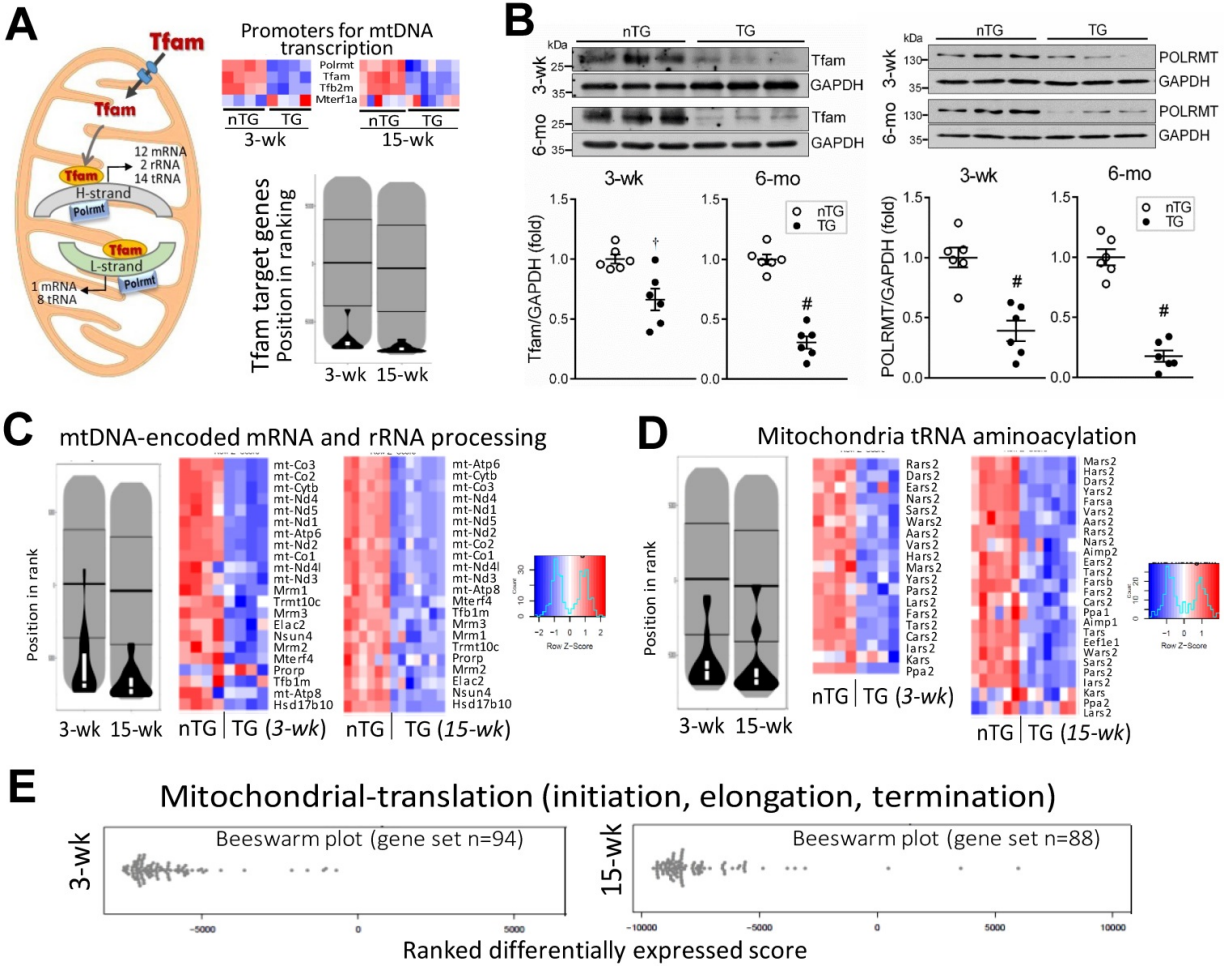
** Downregulation of mitochondrial DNA-encoded genes in TG hearts. A**, Left: diagram depicting the role of Tfam and Polrmt in expression of mtDNA-encoded genes. Heatmap and violin-plot of Reactome analyses showing repression of mitochondria-localized transcriptional promoters in TG versus nTG hearts. **B**, Protein expression of Tfam and Polrmt by immunoblotting of LV myocardium extract of 3-wk and 6-mo mice (n = 6/group). †P < 0.01, ^#^P < 0.001 vs. respective nTG group. Violin-plot and Heatmap of pathway Reactome analyses of expression of mtDNA-encoded mRNAs, nDNA-encoded genes that process premature rRNAs (**C**), and nDNA-encoded synthetases for mitochondrial tRNA aminoacylation (**D**). **E**, Beeswarm plots of changes in TG relative to nTG groups of mitochondrial protein translation genes, in which 86% were mitochondrial ribosome protein (MRP) subunits.

mtDNA transcription is regulated by nDNA-encoded transcription factors. We found, in 3-wk and 15-wk TG hearts, downregulation of mitochondrial polymerase (Polrmt) and transcription factors, Tfam and Tfb2m, but not for mitochondrial transcription termination factor1a (Mterf1a, Figure 8A). Pathway analysis showed profound suppression of Tfam-target genes (Figure 8A). Compared with nTG values, both Tfam and Polrmt at protein level were significantly lower in hearts of TG mice at 3-wk (by 35% and 61%, respectively) and 6-mo (by 69% and 82%, respectively, Figure 8B). Enrichment analysis showed profound suppression in TG versus nTG hearts, of all 13 mtDNA-encoded mRNAs, and nDNA-encoded genes for processing or modifying mtDNA-encoded rRNAs or tRNAs, which are essential for mitochondrial protein transcription (Figure 8C-D). Furthermore, we observed downregulation of nDNA-encoded genes for mitochondrial protein translation, of them 87% were mitoribosome protein (MRP) subunits (Figure 8E).

## Discussion

This study presents a few novel findings from the mouse model with cardiac Hippo signaling activation. *First*, there was comprehensive suppression of mitochondrial gene sets, changes extensively validated directly at mRNA or protein level and indirectly by biochemical assays showing mitochondrial dysfunction. Mechanistically, we found attenuated physical interaction of nuclear YAP/TAZ and TEAD1, suggesting transcriptional inactivation, and repression of mtDNA-encoded genes due to downregulated nDNA-encoded mtDNA transcriptional factors. *Second*, mitochondrial damage with impaired capacity of biogenesis or renewal was indicated by abnormal mitochondrial morphology and changes in marker proteins. *Third*, mitochondrial dysfunction was documented by alterations in tissue contents of ATP, lactate, BCAA, ROS, and mitochondrial lipids, most notably CoQ10 deficiency. Importantly, these changes occur within 2 weeks following transgenic activation of Hippo signaling, implying a causal role of Hippo pathway in mediating mitochondrial damage and DCM.

Independent datasets indicate mitochondrial dysfunction in the Mst1-TG model. In adult TG mouse hearts, ATP content was 50% lower while lactate content increased by 4-fold, which are in keeping with changes in expression of relevant gene sets. In 3-wk TG hearts, whilst the ATP level was unchanged at baseline, an ISO-challenge unmasked the status of energy insufficiency. One contributor to mitochondrial dysfunction is a 50% reduction in the protein level of VDAC1. Being the most abundant outer-mitochondrial membrane (OMM) protein, VDAC1 acts as an interface gate between mitochondrion and cytoplasm for selective transits of a range of molecules like ATP/ADP, NAD/NADH^+^, lipids and ions 37. Upregulation of HIF-1α was evident at mRNA and protein levels and known to occur under hypoxia or in failing myocardium 3, 35. Being a potent metabolic regulator, HIF-1α activation would contribute to metabolic remodeling in Mst1-TG hearts featured by suppressed oxidative phosphorylation but enhanced glycolysis 35, together with upregulated Glut1 as a HIF-1α target 38. Furthermore, contents of BCAAs were markedly higher in TG hearts owing to deficiency in mitochondrial catabolism, a process required for biosynthesis of acetyl-CoA, propionyl-CoA or succinyl-CoA 36. Accumulation of BCAAs, seen in ischemic or failing myocardium 36, 39, might exacerbate metabolic remodeling and inflammatory/fibrotic signaling 36. Another signature abnormality in Mst1-TG hearts is enhanced ROS generation, likely due to uncoupled electron transport and upregulated NOX. Thus, Hippo pathway activation results in comprehensive mitochondrial dysfunction.

In 3-wk and 15-wk TG hearts, there was severe downregulation of nDNA- or mtDNA-encoded mitochondrial genes. Pathway analyses revealed suppression of numerous gene sets for mitochondrial turnover and energy metabolism. Notably, in 3-wk TG hearts, nearly all downregulated gene sets or top-listed DEGs are related to mitochondria, implying a rapid onset of transcriptional disarray following Hippo signaling activation. Our findings by transcriptome were extensively validated by immunoblotting and by a range of biochemical assays, with results showing a high-degree of consistency. Whereas activation of Hippo signaling represses mitochondria-related genes, fibrotic gene sets were simultaneously upregulated, indicating activation of fibrotic signaling at an early phase of DCM. Thus, diverse changes in mitochondrial and fibrotic genes characterize this DCM model. The main signal output of Hippo pathway is through YAP 10, 12. By interacting with transcription factors, e.g. TEADs, YAP acts either as a transcriptional co-activator or co-repressor 9, 40, 41. Studies in YAP-cKO or TEAD1-cKO models showed onset of DCM phenotype within a few weeks after gene deletion 12, 15, 16, 18, a pattern similar to that seen in the Mst1-TG model. This indicates a pivotal role of Hippo/YAP/TEAD1 signaling in the onset of DCM. In keeping with recent reports in TEAD1-cKO mice showing suppressed mitochondrial genes and metabolic dysfunction 16-18, we showed in Mst1-TG hearts a lower nYAP/cYAP ratio together with a reduced physical interaction of nuclear YAP/TAZ and TEAD1, albeit a higher YAP abundance likely due to a compensation to YAP inactivation following Mst1 overexpression. The pivotal role of YAP inactivation in mediating dysregulation of mitochondrial genes was confirmed by our finding, in H9c2 cells, of downregulated mitochondrial marker proteins following YAP gene knockdown.

YAP is known to activate or suppress expression of numerous genes 40, 41, by which the Hippo pathway controls cell fate and organ size. Thus, it is not unexpected to see a high percentage of DEGs in TG hearts, including mitochondrial genes as shown in this study. Notably, nearly all mitochondria-specific proteins tested were downregulated in Mst1-TG hearts, which is due to at least three reasons: *first*, repression of numerous nDNA-encoded genes, *second*, faulty mitochondrial protein import, and *third*, impaired mitochondrial protein synthesis. Expression of mitochondrial protein-importing complex genes is significantly downregulated in TG hearts. Over 99% of mitochondrial proteins, after being synthesized in cytosol, enter into mitochondrion via this protein-import machinery. This is a sophisticated mechanism involving numerous components including translocases of out- and inner-membranes (TOMs, TIMs) with special functions including protein-binding, translocation or modification, and formation of complexes 42. Due apparently to the downregulated transcription promoters, like Tfam and Polrmt, essential for mtDNA transcription,^43^ there was a marked suppression of all mtDNA-encoded mRNAs that direct *in situ* synthesis of proteins of complexes I, III, IV or V. Moreover, significant downregulation of numerous mitoribosomal subunits was evident, changes that would impair synthesis of all mtDNA-encoded proteins 2. Collectively, Hippo signaling activation interferes with nDNA-mtDNA interactions critical to complex mitochondrial function.

The mitochondrion is a lipid-rich organelle 32, and our lipidomics identified three signature lipids: acylcarnitine, CL and CoQ10 32. The most notable change in 3-wk and 6-mo TG hearts is loss of CoQ10, together with profound downregulation of genes for CoQ10 biosynthesis 34. CoQ10 plays a prominent role in redox reactions of the electron-transport chain, and acts as an antioxidant 33. There are studies reporting reduction in blood levels of CoQ10 and beneficial effects of CoQ10 supplementation to patients with cardiomyopathy and HF 33, 44. Our findings support CoQ10 supplementation therapy of heart disease, but also indicate a mechanistic link between Hippo signaling and CoQ10 deficiency. Several studies reported higher plasma acylcarnitine levels in HF patients 45, which was regarded as due to cardiac leakage into blood circulation. Our DCM model had elevated tissue and plasma levels of acylcarnitine, indicative of blockade of acylcarnitine/carnitine trafficking into mitochondria. CL is another mitochondria-signature lipid accounting for 15-20% of total phospholipids 32, 46. By interacting with mitochondrial membrane proteins, CL participates in assembly and stabilization of super-complexes, the electrochemical gradient essential for ATP synthesis, cristae formation and dynamic turnover 32, 46. Reduced content and/or remodeling of CL have been reported in the failing heart 47. In Mst1-TG hearts, whereas CL content was largely maintained, there were overt changes in its 4 fatty-acyl-chains that determine its degree of diversity and functional flexibility 32. Certain changes in CL acyl-chain patterns have been linked to mitochondrial enzymatic dysfunction, like loss of linoleic acid (18:2) predominant CL or replacement of 18:2 with docosahexaenoic acid (22:6) 47-49. Mst1-TG hearts had increased CL with predominately saturated/monounsaturated fatty acids but reduced CL with tetra-18:2 or tri-18:2 (-26%) or with 22:6 (-49%). Fatty-acyl-chains of CL or its precursor monolysocardiolipin (MLCL) are modified by mitochondrial matrix-localized enzymatic machinery 32, 46. Our transcriptome revealed diverse changes in the expression of these enzymes.

Mitochondrial structural abnormalities in TG hearts were evident both in quality and quantity. Faulty mitochondrial turnover was strongly indicated by downregulated gene-sets and low levels of marker proteins for biogenesis (e.g. PGC-1α, NRF1), fusion (e.g. Mfn, Opa) or mitophagy, a process pivotal in mitochondrial quality control and renewal,^2^ whilst fission proteins (e.g. Fis1, p53) were increased. Enhanced AMPKα signaling in TG hearts might be compensatory in promoting biogenesis. Regulation by YAP of mitochondrial turnover was indicated by a recent study showing that cells with YAP overexpression had larger mitochondria due to enhanced fusion while YAP inactivation stimulates mitochondrial fission 50.

Does Hippo signaling activation/YAP inactivation directly cause mitochondrial damage and DCM in the Mst1-TG model? We attempted to address this question by simultaneously studying 3-wk and adult Mst1-TG mice, given that transgene expression driven by αMHC promoter becomes active from week-1 after birth 14, 31. Adult TG mice displayed severe cardiac dysfunction, chamber dilatation, severe fibrosis and organ congestion, evidence of advanced HF 13, 14. Relative to adult counterparts, TG mice at 3-wks had comparable downregulation of mitochondrial genes/proteins, but were less severe in mitochondrial morphological abnormalities, unchanged tissue content of ATP and lactate at baseline, and absence of either LV dilatation or fibrosis. These findings suggest an early phase of DCM at this age. Indeed, contractile dysfunction of LV and RV was the main abnormality by echocardiography in young TG mice, very likely secondary to mitochondrial dysfunction. Our findings from the 3-wk TG model is in keeping with published studies, showing that inducible gene knockout of YAP or TEAD1 leads to onset of DCM within a few weeks 12, 15, 16, 18, and that TEAD1-KO results in profound mitochondrial dysfunction 16, 17. Collectively, Hippo activation (e.g. Mst1-TG) or YAP/TEAD1 inactivation mediates cardiac mitochondrial dysfunction 16, 17, that constitutes a causal mechanism in *de novo* onset of DCM. Recent studies implicated regulation by the Hippo pathway of fibrosis signaling 7, 12, 13, 15, 40, 41. Here we provided direct evidence for the Hippo pathway in mediating cardiac fibrotic signaling. Several profibrotic factors, like galectin-3 and CTGF are known as YAP-target genes, and their transcription in the heart is enhanced upon YAP inactivation 9, 13, 40.

A few limitations of this study deserve comment. *First*, we only studied a single TG model without using alternative *in vivo* model(s) for validating our findings. The causal role of Hippo-YAP-mitochondrion axis in DCM pathogenesis is established by our findings on 3-wk Mst1-TG mice, and studies of inducible TG or KO models would provide additional confirmation. Nevertheless, our findings, including downregulation of mitochondrial proteins in cells following YAP knockdown, have furthered the recent studies on TEAD1-cKO model 16, 17, by showing that it is Hippo signaling that represses YAP/TAZ-TEAD1 transcriptional activity. *Second*, whereas we observed significant changes in plasma levels of CoQ10, acylcarnitine and BCAA in DCM mice, further validation including clinical study is absent, which is essential for establishing a panel of biomarkers for cardiac metabolic remodeling.

In conclusion, we documented that Hippo signaling activation/YAP-TEAD1 inactivation leads to mitochondrial damage and dysfunction by repressing numerous mitochondrial genes. Our findings imply Hippo signaling as a therapeutic target for protecting the mitochondrion in cardiomyopathy and HF.

## Supplementary Material

Supplementary figures and tables.

## Figures and Tables

**Table 1:** Echocardiographic parameters of 4-week-old nTG and TG male mice

	nTG	TG	P-value
Number	6	4	
Body weight (g)	16.5 ± 0.4	15.4 ± 0.9	0.216
**B-mode Loop image**			
Heart rate (beats/min)	518 ± 12	445 ± 34	0.042
LV Area-Diastole (mm^2^)	18.1 ± 0.9	18.7 ± 0.7	0.649
LV Area-Systole (mm^2^)	12.0 ± 0.6	14.7 ± 0.9	0.027
Area change (%)	34 ± 1	21 ± 2	0.000
LV Volume-diastole (μL)	41 ± 4	45 ± 2	0.467
LV Volume-systole (μL)	21 ± 2	30 ± 2	0.020
Stroke volume (μL)	20 ± 2	15 ± 1	0.047
LV ejection fraction (%)	49 ± 2	34 ± 3	0.001
Cardiac output (ml/min)	10.5 ± 0.9	6.6 ± 0.4	0.012
Aorta dimension (mm)	1.06 ± 0.04	0.93 ± 0.07	0.130
Left atria dimension (mm)	1.90 ± 0.15	3.23 ± 0.15	0.000
Left atria/Aorta ratio	1.79 ± 0.13	3.56 ± 0.38	0.001
RV Dimension-diastole (mm)	1.65 ± 0.10	2.54 ± 0.12	0.000
RV Dimension-systole (mm)	1.12 ± 0.10	1.92 ± 0.14	0.002
RV fractional shortening (%)	32 ± 3	25 ± 2	0.111
**M-mode image**			
LV Dimension-diastole (mm)	3.22 ± 0.20	3.20 ± 0.18	0.941
LV Dimension-systole (mm)	2.01 ± 0.14	2.59 ± 0.18	0.032
Fractional shortening (%)	37 ± 2	19 ± 2	0.001
Anterior WT-diastole (mm)	0.66 ± 0.04	0.62 ± 0.07	0.611
Posterior WT-diastole (mm)	0.62 ± 0.04	0.72 ± 0.05	0.186
Echo LV mass (mg)	61 ± 3	64 ± 4	0.574

LV: left ventricle; RV: right ventricle; WT: wall thickness.

## Abbreviations

AC: acyl-carnitine
BCAA: branched chain amino acids
CL: cardiolipin
CoQ10: coenzyme Q10
DCM: dilated cardiomyopathy
DEGs: differentially expressed genes
FS: fractional shortening
HF: heart failure
HIF-1α: hypoxia inducible factor-1α
IMM: inner mitochondrial membrane
ISO: isoproterenol
LV: left ventricular or ventricle
KO: knockout
mtDNA: mitochondrial DNA
Mst1: mammalian sterile-20-like kinase-1
nDNA: nuclear DNA
Polrmt: polymerase mitochondrial
OMM: outer mitochondrial membrane
ROS: reactive oxygen species
RV: right ventricular or ventricle
TAZ: transcriptional Co-activator with PDZ-binding Motif
TCA: tri-carboxylic acid
TEAD1: TEA-domain family member1
Tfam: transcription factor A mitochondrial
TG: transgenic
YAP: yes-associated protein
