# Supplementary material for: Activation of Hippo signaling pathway mediates mitochondria dysfunction and dilated cardiomyopathy in mice
Source: Theranostics. 2021 Aug 21;11(18):8993–9008. doi: 10.7150/thno.62302 (PMC8419046; doi:10.7150/thno.62302)

Theranostics_ID-62302b2: **Online Supplementary Material**

Activation of the Hippo signalling pathway mediates mitochondria dysfunction and dilated cardiomyopathy in mice

Wei Wu, Mark Ziemann, Kevin Huynh, Gang She, Zhen-Da Pang, Zhang Yi, Thy Duong, Helen Kiriazis, Tian-Tian Pu, Ru-Yue Bai, Ming-Xia Chen, Junichi Sadoshima, Jing-Jing Li, Yu Zhang, Xiu-Ling Deng, Peter J. Meikle, Xiao-Jun Du

Correspondence: XJ Du, email: xiao-jun.du@baker.edu.au

SUPPLEMENTARY MATERIALS

Expanded Methods

Table S1. Source and characteristics of antibodies used for immunoblotting

Table S2. Oligonucleotide sequences of mouse primers for gene expression by RT-PCR

Table S3. Full list of abbreviations use in figures

Figure S1. Cardiomyopathy phenotype of 3-wk Mst1-TG mice

Figure S2. Cardiac transcriptome by RNA sequencing

Figure S3. Downregulation of gene sets of mitochondrial biosynthesis, assembly and turnover in TG hearts

Figure S4. Downregulation of gene sets of mitochondrial metabolism

Figure S5. Downregulation of mitochondrial protein import genes in Mst1-TG hearts by RNA-sequencing

Figure S6. Upregulation in fibrotic pathway gene-sets in 3-wk and 15-wk Mst1-TG mouse hearts and myocardial fibrosis in adult TG hearts

Figure S7. Lack of changes in certain pathways by transcriptome of 3-wk and 15-wk Mst1-TG mouse hearts

Figure S8. Changes in YAP-target genes in TG heart by transcriptome.

RNA sequencing data were uploaded to the Gene Expression Omnibus (GSE106201)

Supplementary Materials

Expanded Methods

Transmission electron microscopy (EM) and quantification

Mitochondrial ultrastructure was studied in freshly collected LV specimens by EM (3 LV samples per age per genotype). Samples were dissected into 1 mm^3 blocks, immediately fixed with 2.5% glutaraldehyde buffer (2 h at 37ºC), and then post-fixed in 1% OsO_4 (2 h at 4ºC). The samples were dehydrated in a graded series of alcohol and then exposed to propylene oxide for infiltration of the embedding medium, Epon 812 resin in 0.1 mol/L sodium phosphate buffer. Using LKB-V/Leica UC7 ultramicrotome (LKB-V/NOVA, Sweden and Leica, Germany), the embedded blocks were cut into sections (50-70 nm). Sections were stained with acidified uranyl acetate, followed by a modification of Sato's triple lead stain, and viewed with a transmission electron microscope (TEM; H-7650; Hitachi, Tokyo, Japan). For each sample, images from randomly chosen fields were obtained at magnifications of ×4,000, ×10,000 and ×30,000, respectively (6-10 images at each magnification per heart sample). The images (×10,000 magnification) were analysed, in a blinded fashion, using ImageJ software on at least 8 images per heart. Area of individual mitochondria was manually measured and average used. The sum of all measurements of mitochondria size per image represented the total area by mitochondria per image field. Mitochondria with complete or incomplete outer membrane were individually observed and counted to obtain percentage of mitochondria with incomplete membrane. Sarcomere length was determined in 8-10 images (×10,000 magnification) by drawing a line parallel to myofibril orientation across over 5-10 sarcomeres. Approximately 30 sarcomeres were measured from one image and the averages from all images calculated for comparison.

Mitochondrial lipid and amino acid profiling

Tissue homogenization and lipid extraction. Tissue samples were homogenized using a sonicator probe in ice cold PBS. Lipids were extracted from homogenized tissue as previously described.[1] In brief, tissue or plasma was mixed with 10× volume of butanol:methanol (1:1) with 10 mM ammonium formate containing a mixture of internal standards. Samples were vortexed thoroughly and set in a sonicator bath for 1 hour at room temperature. Samples were then centrifuged (14,000*g*, 10 min, 20 ºC) and the supernatant was transferred into sample vials with glass inserts for mass spectrometry analysis.

Liquid chromatography mass spectrometry (LC/MS/MS). The detailed lipidomics and assay conditions have been reported previously.[2] Briefly, lipidomic analysis was performed by LC ESI-MS/MS using an Agilent 1290 liquid chromatography system and an Agilent 6490 QQQ mass spectrometer. We employed source conditions identical to those previously described.[2] Liquid chromatography was performed on a Zorbax C18, 1.8 µm, 50× 2.1 mm column (Agilent Technologies). Solvents A and B consisted of water, acetonitrile and isopropanol in the ratio (50:30:20) and (90:9:1) respectively, both containing 10 mM ammonium formate with solvents B also containing 5 µM medronic acid. Gradient conditions were as previously described [1, 2]. Columns were heated to 45 °C and the auto-sampler regulated to 25 °C.

Analysis of mitochondria-rich lipid species. Quantification of lipid species was determined by comparison to the relevant internal standards. In the present study that was focused on mitochondria, only 3 lipids that are largely or entirely localized in mitochondria were analysed, i.e. acyl-carnitine (AC), cardiolipin (CL) and ubiquinone (or coenzyme Q10, CoQ10). Lipid characterization and quantification were conducted as we previously described in detail [1, 2]. Alterations in the species of the three lipids were expressed as relative change to that of nTG control.

Amino acid analysis of heart and plasma. The extracted myocardial samples were further analysed for specific amino acids using a separate targeted HILIC-MS/MS method. An Acquity UPLC BEH Amide 1.7 µm 2.1 × 100 mm column (Waters) was used. In this assay Solvent A comprises of 50% acetonitrile in water, whereas Solvent B comprises of 95% acetonitrile in water, both with 10 mM ammonium formate. Chromatography was used to separate out analytes prior to mass spectrometetry analysis. Starting at 100% B, this was held for 1 minute before a linear gradient to 0% B over 5 minutes. 1 minute was then spent holding 0% B before switching to 100% B over 0.1 minute, then held at 100% B for an additional 2.9 minutes for equilibration. Total run time was 10 minutes per sample. Results were normalised to a spike internal standard (L-Leucine-5,5,5-d3, Sigma Aldrich).

High performance liquid chromatography (HPLC) assay of ubiquinone: Tissue content of ubiquinone in LVs of 3-wk-old mice was determined using HPLC. LV tissues (20 mg) were homogenized in 2 ml extract (N-hexane: ethanol = 8:1), centrifuged (3,000 rpm for 5 min), and supernatant was harvested. This process was repeated 3 times and the final supernatant was evaporated to dryness in a vacuum centrifuged concentrator (1500 rpm, 37°C, 50 min). Sample was redissolved in pure ethanol (100 µL) and assayed using SHIMADZU HPLC system (LC-2030C 3D, mobile phase: 3:7 methanol:ethanol 1 ml/min). Ubiquinone was identified by an internal standard and the amount quantified using ubidecarenone (Sigma, Lot# LRAC3727) derived standard curve.

References

1. Tham YK, Bernardo BC, Huynh K, Ooi JYY, Gao XM, Kiriazis H, et al. Lipidomic Profiles of the Heart and Circulation in Response to Exercise versus Cardiac Pathology: A Resource of Potential Biomarkers and Drug Targets. Cell Rep. 2018;24:2757-2772.
2. Huynh K, Barlow CK, Jayawardana KS, Weir JM, Mellett NA, Cinel M, et al. High-Throughput Plasma Lipidomics: Detailed Mapping of the Associations with Cardiometabolic Risk Factors. Cell Chem Biol. 2019;26:71-84 e4.

Quantitative real-time PCR

RNA was extracted from LV tissue using RNAiso PLUS (Takara Bio, Japan, Code No. 9108/9109). RNA (1 µg) was used for cDNA synthesis. Quantitative PCR was performed by SYBR-based RT-PCR assays using PrimeScript^{TM} reagent kit with gDNA Eraser (Perfect Real-Time) (Takara, Code No: RR047A), and TB Gree^{TM} Premix Ex Tag^{TM} II Tli RNAaseH Plus (Takara, Code No. RR820A). Table S1 lists target genes examined and sequences of primers (Sangon Biotech, Shanghai, China). Quantitative real-time PCR reaction was performed using Bio-Rad CFX96 Real-time PCR Detection System with the following two step procedures: Step-1: 95 °C for 30 sec, and Step-2 (PCR reaction): 40 cycles of amplification at 95 °C for 5 sec and 60 °C for 30 sec. The expression of target genes was

normalized to that of GAPDH using the method of $2^{-\Delta\Delta ct}$, and presented relative to that of the nTG value.

YAP silencing by small interfering RNA in H9c2 cells.

YAP gene knockdown was performed in rat cardiomyoblasts (H9c2) using small interfering RNA (siRNA, GenePharma, Shanghai), according to manufacturer's instructions. Cells were cultured in 6-well plates with standard antibacterial-free medium. To make siRNA delivery medium, 2.5 µL lipofectamine 2000 (Invitrogen 11668019) was added in 250 µL Opti-MEM (Gibco, 11058021) and mixed for 5 min, followed by addition of specific rat YAP-siRNA (F: 5-GGA GAA GUU UAC UAC AUA ATT-3; R: 5-UUA UGU AGU AAA CUU CUC CTT-3) or non-targeting control siRNA (10 nM each) with 20 min allowed for full mixing. After reaching 40-50% confluence, cells were incubated with the siRNA delivery medium (0.5 mL per well containing 1.5 mL standard medium). Six hours afterward, the delivery medium was replaced. After incubation for another 48 h, cells were harvested and protein was extracted for immunoblotting assay.

Table S1. Oligonucleotide sequences of mouse primers for gene expression by RT-PCR

Gene name	Primer Sequence (5'—3')
Ctgf	F: GACCCAACTATGATGCGAGCC
	R: ACTTAGCCCTGTATGTCTTCACA
Lgals3	F: AACACGAAGCAGGACAATAACTGG
	R: GCAGTAGGTGAGCATCGTTGAC
IL6	F: GGTACATCCTCGACGGCATCT
	R: GTGCCTCTTTGCTGCTTTCAC
Axl	F: ATGCCAGTCAAGTGGATTGCT
	R: CACACATCGCTCTTGCTGGT
Ankrd1	F: GAGACACCCCACTGCATGAT
	R: TTCCCAGCACAGTTCTTGACC
Ndufa12	F: ACCCTCCGACGACTAATCCA
	R: GTGTCGAAGGTGGAACCCAT
Ndufab1	F: CTGAGGGAATCCGGAGGAGA
	R: GTCTAACGTCAGTGGGGGTG
Ndufs6	F: CCTGGAGCGATTCTGGGATAACTT
	R: TGGGCTTCGAGCTAACAATGGTGT
Atp5e	F: GAGGCTACTCTGAAGCGACC
	R: ACCGGATGTAGCTGAGTCCA
Txn2	F: GCTAGAGAAGATGGTCGCCAAGCAGCA
	R: TCCTCGTCCTTGATCCCCACAAACTTG
Ndufa3	F: TGTCTGGGGCCTCGCTATAA
	R: CAGGCATGTTCCCGTCATCT
Gapdh	F: TGAAGCAGGCATCTGAGGG
	R: CGAAGGTGGAAGAGTGGGAG

Abbreviations: please refer to online supplemental Table 3.

Table S2. Source and characteristics of antibodies used for immunoblotting

Protein	supplier	Lot number	concentration	MW (kDa)
Mst1	CST	#3682	1:1000	59
p-Yap	CST	#4911	1:1000	69
Yap	CST	#12395	1:1000	69
PGC-1α	proteintech	66369	1:1000	100
NRF1	CST	# 46743S	1:1000	68
AMPKα	CST	#2532	1:1000	62
OPA1	CST	# 80471S	1:3000	80-100
OPA3	proteintech	15638-1-AP	1:1000	20
Mfn1	abcam	ab104274	1:1000	84
DRP1	CST	# 8570S	1:1000	82
Fis1	abcam	ab71498	1:1000	17
p53	abcam	ab26	1:1000	53
LC3A	CST	# 4599S	1:1000	14,16
Pink1	abcam	ab23707	1:500	66
Bnip3	abcam	ab10433	1:1000	30
MT-ND1	abcam	ab181848	1:5000	36
SDHA	CST	#11998	1:3000	70
COXIV	proteintech	11242-1-AP	1:4000	17
OGDH	abcam	ab137773	1:5000	116
PDH	CST	#3205	1:3000	43
Bax	CST	#2772S	1:1000	20
Bcl-2	CST	#3498S	1:1000	26
Hif-1α	CST	#36169	1:1000	120
VDAC1	proteintech	10866-1-AP	1:4000	31
TFAM	abcam	ab131607	1:2000	28
Anti-MYH6	abcam	ab207926	1:3000	223
Anti-MYH7B	abcam	ab172967	1:3000	221
GAPDH	proteintech	10494-1-AP	1:4000	36
CTGF	proteintech	23936-1	1:500	38
Yap1	santa	sc-376830	1:500	70
Taz	CST	#84185	1:1000	55
Tead1	santa	sc-393976	1:500	50
Tead1	CST	#12292	1:1000	50
Lamin B1	Proteintech	66095-1-Ig	1:3000	66
Galectin-3	abcam	ab2785	1:1000	30
IL-6	CST	12912T	1:1000	24
NDUFA12	abcam	Ab192617	1:4000	17
NDUFAB1	abcam	Ab181021	1:1000	12
NDUFS6	abcam	Ab195808	1:1000	14
Txn2	abcam	Ab185544	1:10000	12
POLRmt	Invitrogen	PA5-28129	1:1000	130
IgG	Beyotime	A7028		

Table S3. Full list of abbreviations used in Figures

Fig 1 Abbreviations	Full term
TG, nTG	Transgenic, non-transgenic
Mst1	mammalian sterile-20 like kinase 1
MHC	myosin heavy chain
YAP	yes-associated protein
GAPHD	glyceraldehyde-3-phosphate dehydrogenase
LV	left ventricle or left ventricular
LA	left atrium
Ao	aorta

Fig 4 Abbreviations	Full term
PGC-1α	peroxisome proliferator-activated receptor γ coactivator 1-α
NRF1	nuclear respiratory factor 1
AMPKα	AMP-activated protein kinase α
Mfn1	mitofusin 1
OPA1/3	optic atrophy 1/3, mitochondrial dynamin-related protein
Drp1	dynamin-related protein 1
Fis1	fission, mitochondrial 1
Bnip3	BCL2 interacting protein 3
Pink1	PTEN induced kinase 1
LC3II/I	microtubule associated protein 1 light chain 3 alpha
MT-ND1	NADH dehydrogenase, subunit 1 (complex I)
SDHA	succinate dehydrogenase complex flavoprotein subunit A
COX IV	cytochrome c oxidase subunit 4
OGDH	oxoglutarate dehydrogenase
PDH	pyruvate dehydrogenase
Bcl-2	BCL2 apoptosis regulator
Bax	BCL2 associated X, apoptosis regulator
VDAC1	voltage-dependent anion channel 1
GAPDH	glyceraldehyde-3-phosphate dehydrogenase

Fig 5 Abbreviations	Full term
AC	acylcarnitine
CL	cardiolipin
Coq2-10	hydroxybenzoate polyprenyltransferases 2-10, mitochondrial
Pdss1/2	Decaprenyl-diphosphate synthase subunit 1/2
CPT1/2	Carnitine palmitoyltransferases
CACT	Carnitine/acylcarnitine translocase
TAZ	transcriptional coactivator with PDZ-binding motif
HADHa/b	hydroxyacyl-CoA dehydrogenase trifunctional multienzyme complex
LCLAT1	lysocardiolipin acyltransferase 1
CRLS1	cardiolipin synthase 1
PLA2G6	phospholipase A2 group VI
TAMM41	TAM41 mitochondrial translocator assembly and maintenance homolog
PGS1	phosphatidylglycerophosphate synthase 1
HSD10	fibrous sheath interacting protein 1
PLD6	phospholipase D family member 6

Fig 6 Abbreviations	Full term
Glut1	glucose transporter1
HIF-1α	hypoxia inducible factor-1α
BCAA	Branched-chain amino acid
BCAT2	branched-chain-amino-acid aminotransferase
BCKDH	Branched-chain keto acid dehydrogenase E1
BCKDK	Branched-chain keto acid dehydrogenase kinase
PPM1K	protein phosphatase 1K
Mccc1/2	methylcrotonoyl-CoA carboxylase
IVD	isovaleryl-CoA dehydrogenase
ACAT1	acetyl-CoA acetyltransferase 1
Acad	acyl-CoA dehydrogenase short chain
Pcca	propionyl-CoA carboxylase subunit alpha
Mtr	5-methyltetrahydrofolate-homocysteine methyltransferase
NOX2/4	NADPH oxidases2/4

Fig 7 Abbreviations	Full term
IP	immunoprecipitation
TAZ	Tafazzin
CTGF	Connective tissue growth factor
Lgala3 or Gal3	Galectin-3
Ankrd1	Ankyrin repeat domain 1
Axl	AXL receptor tyrosine kinase
Hsf1/2	Heat shock transcription factor 1/2
Txn2	Thioredoxin, mitochondrial
Ndufs	NADH:ubiquinone oxidoreductase subunits
Apt5e	ATP synthase 5E
IL-6	Interleukin-6

Fig 8 Abbreviations	Full term
mtDNA	Mitochondrial DNA
Tfam	transcription factor A, mitochondrial
Polrmt	RNA polymerase, mitochondrial
Tfb2m	transcription factor B2, mitochondrial
Mterf1a	mitochondrial transcription termination factor 1a

SUPPLEMENTAL FIGURES

Figure S1.

Dilated cardiomyopathy phenotype of 3-wk Mst1-TG mice. Images of lungs and the liver from nTG and Mst1-TG mice at 3 weeks (A) in comparison to that of 6-mo-old counterparts (B). C, body weight-normalized organ weights of 3-wk-old male and female mice. Pulmonary and hepatic congestion in TG mice indicated presence whole heart failure. *P < 0.05, †P<0.01, #P < 0.001 vs. nTG group.

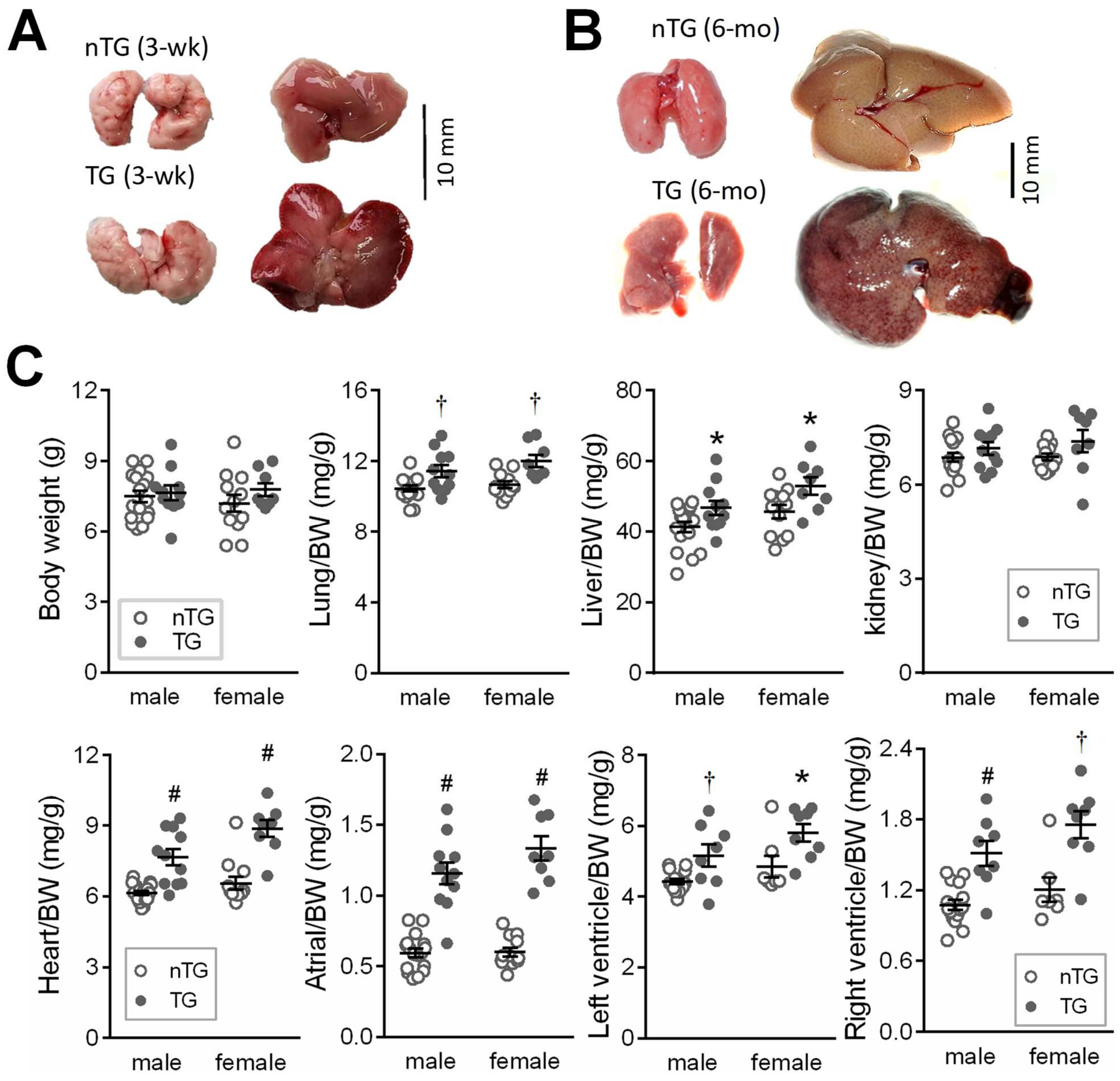

Figure S2.

Cardiac transcriptome by RNA sequencing.

RNA sequencing data were collected from LV tissues of mice aged at 3-wk (n = 4/genotype) and 15-wk (n = 6 for nTG and n = 7 for TG). We generated 25 M (3-wk) or 38.6 M (15-wk) reads/sample, of which 83.3% of reads were uniquely mapped (SD 2.1%). An average of 24.0 M reads was assigned to genes (SD 3.2 M). **A**, Venn diagrams showing number of genes detected in 3-wk and 15-wk TG hearts (left), and number of differentially expressed genes (DEGs, FDR<0.05) due to Mst1-overexpression in both age groups (right). **B**, Volcano plots of DEGs of 3-wk and 15-wk TG mice relative to respective nTGs. Red dots denote genes with FDR < 0.05). For clarity reason, only DEGs are presented. **C**, Venn diagram of number of genes showing significant changes due to Mst1 overexpression in both age groups. **D**, Filled contour plot of DEGs in TG hearts showing a positive correlation between 3-wk and 15-wk TG groups.

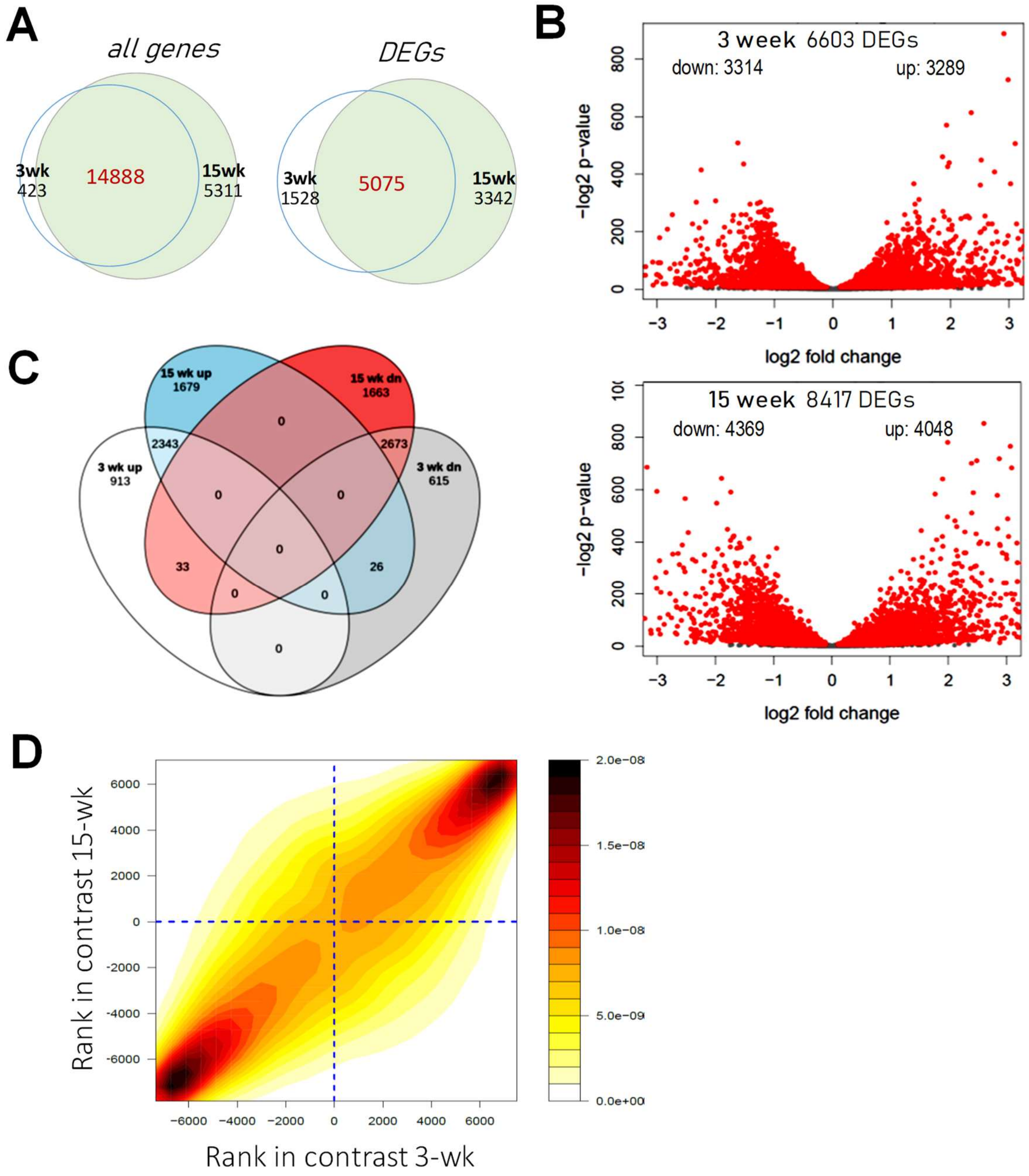

Figure S3.

Downregulation of gene sets of mitochondrial biosynthesis, assembly and turnover in TG hearts.

Filled contour plots showing that 3-wk and 15-wk TG hearts exhibited similar changes in the down-regulation of gene sets related to mitochondria biosynthesis, assembly, turnover, cristae formation, protein import and metabolism of cofactors.

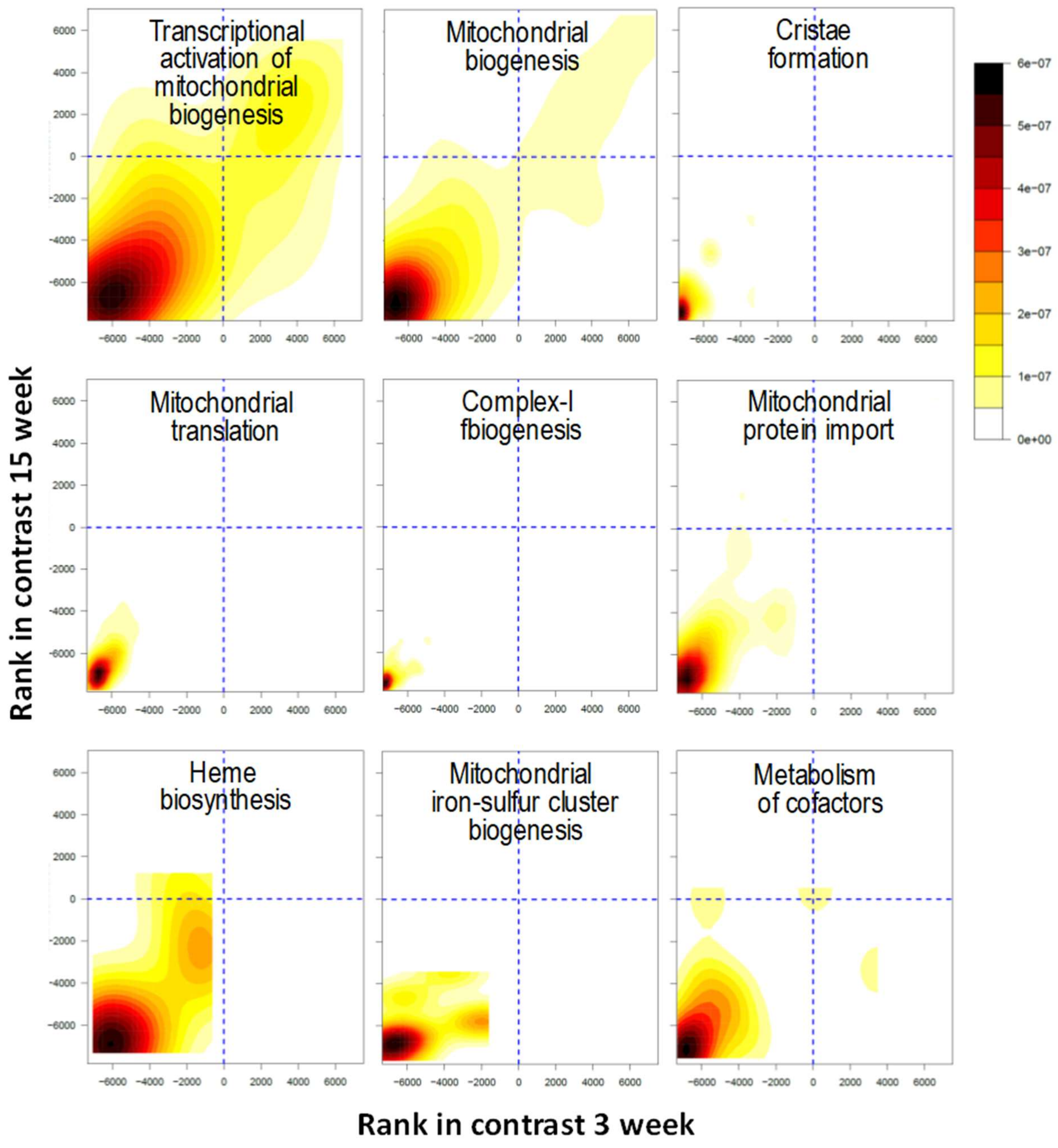

Figure S4.

Downregulation of gene-sets of mitochondrial metabolism. Filled contour plots showing that 3-wk and 15-wk TG mouse hearts exhibited consistent down-regulation of gene-sets related to mitochondria metabolism.

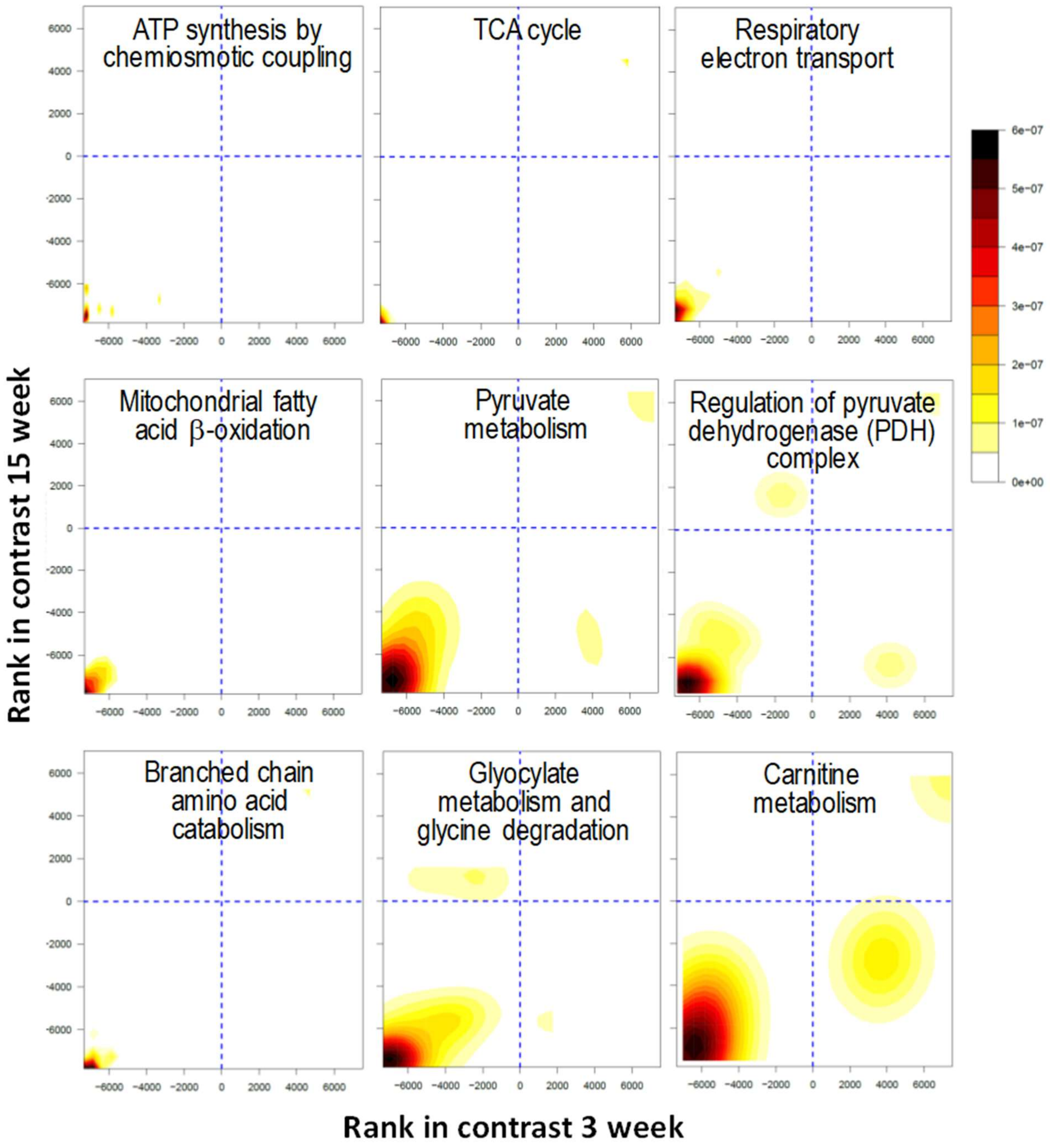

Figure S5.

Downregulation of mitochondrial protein import genes in Mst1-TG hearts by RNAsequencing.

Heatmap for mitochondrial protein import genes (set size n = 57) in 3-wk and 15-wk Mst1-TG hearts. Among the gene-sets were numerous members of translocases of outer-membrane (TOMs) or translocases of inner-membrane (TIM) that mediate inward transportation of cytosolic proteins into mitochondria. Group size: n = 4/group for 3-wk and n = 6-7/group for 15-wk group.

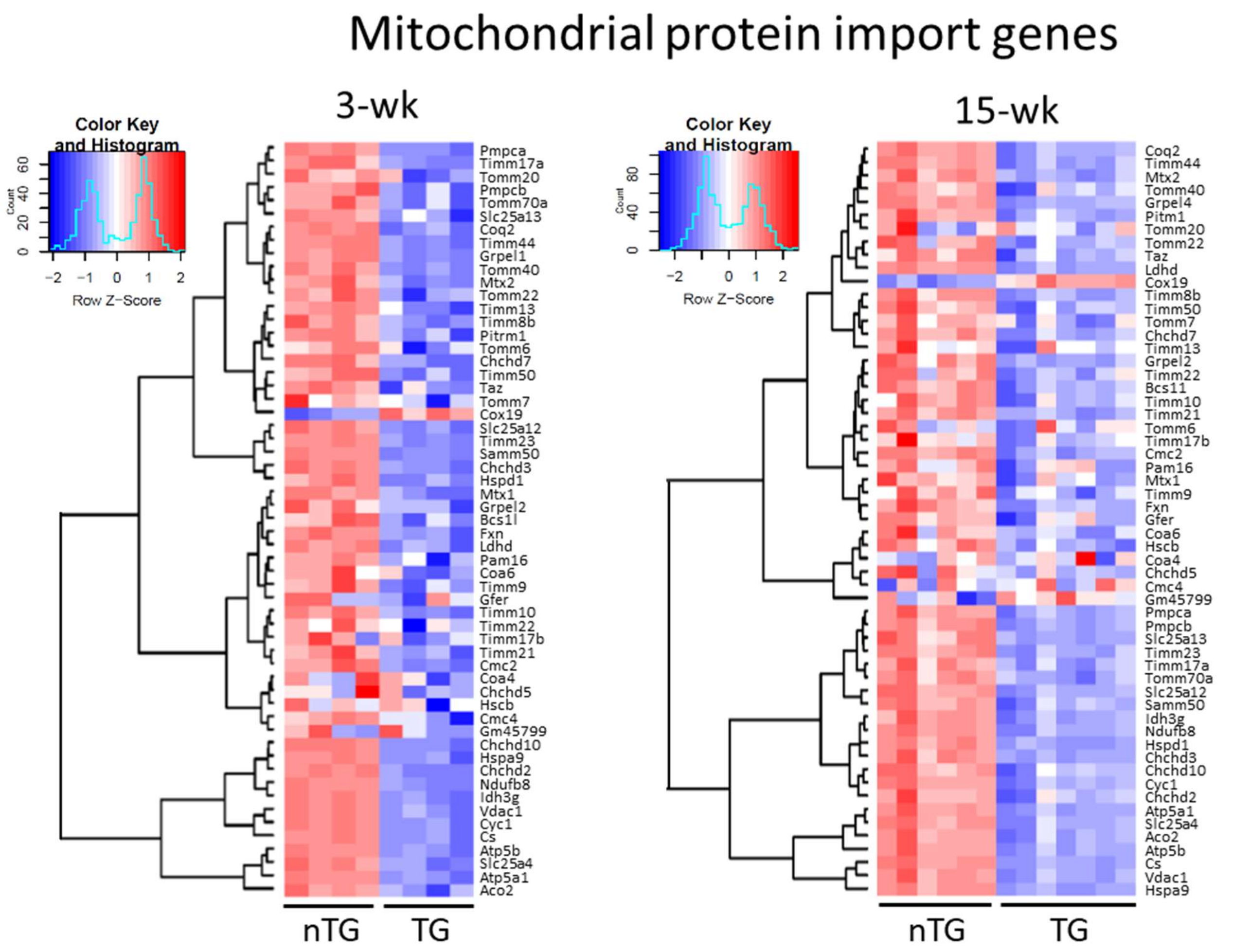

Figure S6.

Upregulation in fibrotic pathway gene-sets in 3-wk and 15-wk Mst1-TG mouse hearts and myocardial fibrosis in adult TG hearts.

A, Filled contour plots showing similarity in 3-wk (n = 4/group) and 15-wk (n = 6-7/group) TG relative to nTG hearts in upregulated expression of gene-sets related to extracellular matrix formation/turnover and fibrogenesis. **B**, Representative histology of nTG and TG hearts (left ventricle, LV) with Masson trichrome staining for collagen in blue colour. Note that significant interstitial fibrosis (in blue) of LV myocardium was evident in 6-month-old but not in 3-wk-old TG mice.

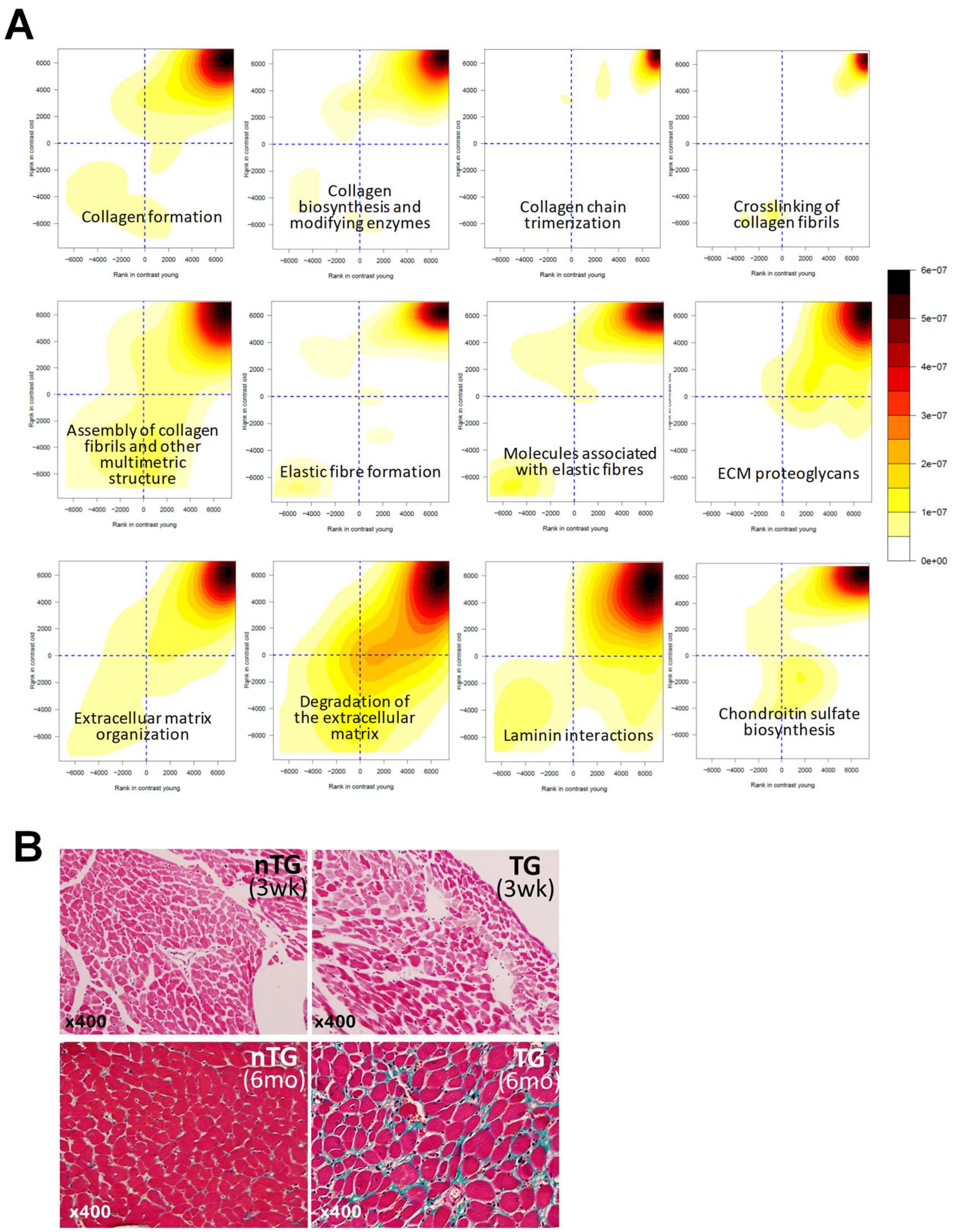

Figure S7.

Lack of changes in certain pathways by transcriptome of 3-wk and 15-wk Mst1-TG mouse hearts.

Heatmap for selected pathways (cellular responses to stress, hemostasis, apoptosis, signal transduction, cardiac conduction, muscle contraction) in Mst1-TG hearts. Note the lack of differences between nTG and TG groups in expression pattern of these gene sets.

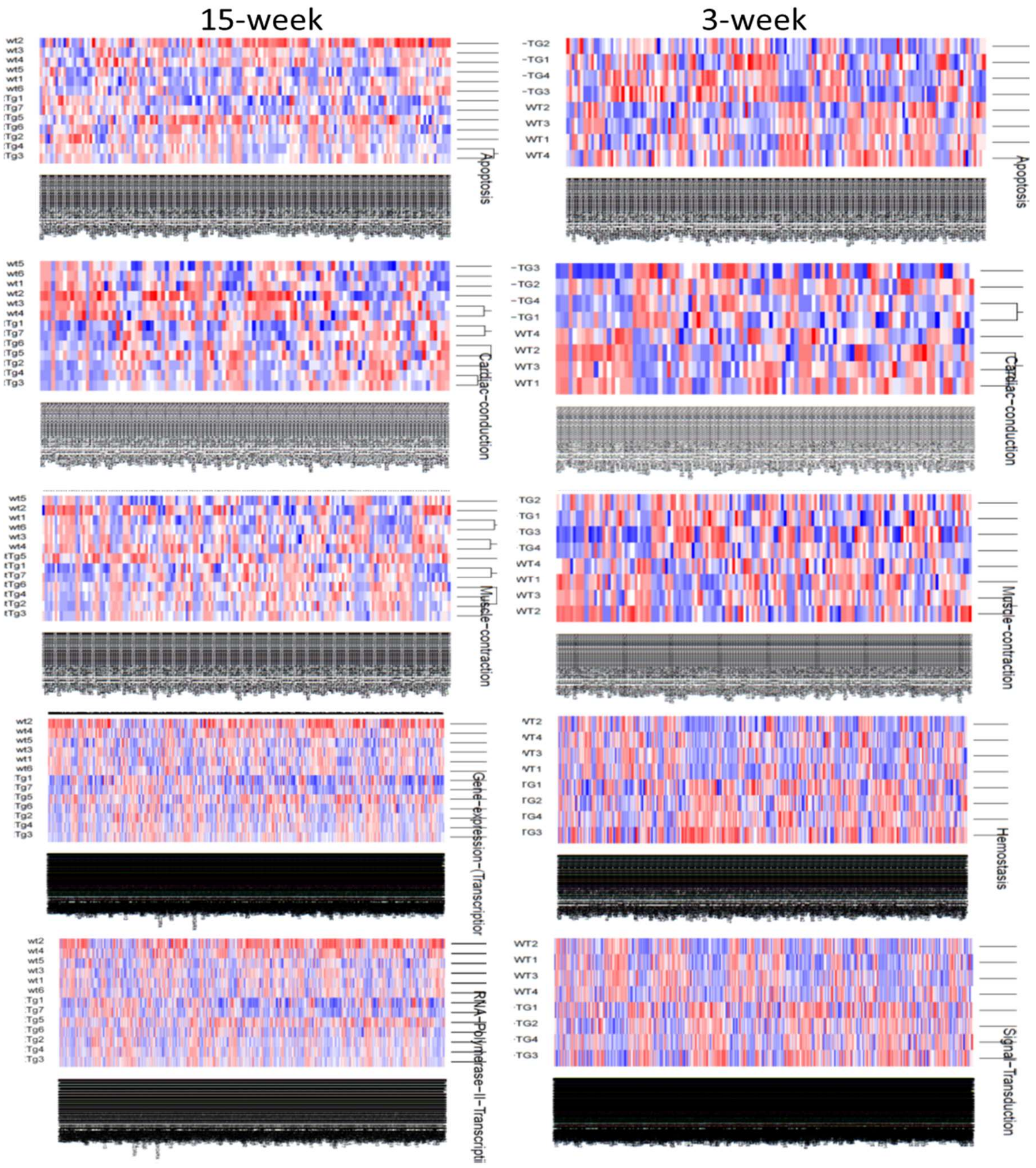

Figure S8.

Changes in YAP-target genes in TG heart by transcriptome.

A, Volcano plots showing up- and down-regulated YAP-target genes in 3-wk and 15-wk TG relative to respective nTG hearts. Red dots denote genes with FDR < 0.05. **B**, Venn diagram of downregulated genes in both TG groups. Bar graph depicting the number of downregulated mitochondrial genes as percentages of total downregulated YAP-target genes.

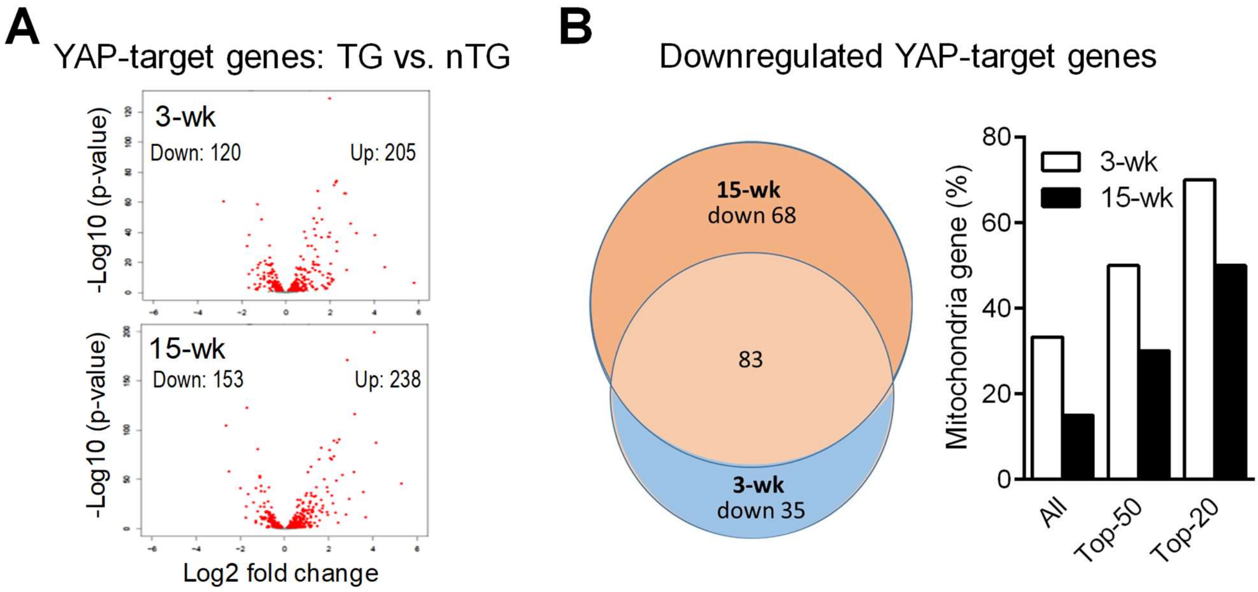

Supplement: Supplementary file 1 — Supplementary figures and tables. [file thnov11p8993s1.pdf]
